# Multistate matrix population model to assess the contributions and impacts on population abundance of domestic cats in urban areas including owned cats, unowned cats, and cats in shelters

**DOI:** 10.1371/journal.pone.0192139

**Published:** 2018-02-28

**Authors:** D. T. Tyler Flockhart, Jason B. Coe

**Affiliations:** 1 Department of Population Medicine, Ontario Veterinary College, University of Guelph, Guelph, Ontario, Canada; 2 Department of Integrative Biology, University of Guelph, Guelph, Ontario, Canada; Auburn University, UNITED STATES

## Abstract

Concerns over cat homelessness, over-taxed animal shelters, public health risks, and environmental impacts has raised attention on urban-cat populations. To truly understand cat population dynamics, the collective population of owned cats, unowned cats, and cats in the shelter system must be considered simultaneously because each subpopulation contributes differently to the overall population of cats in a community (e.g., differences in neuter rates, differences in impacts on wildlife) and cats move among categories through human interventions (e.g., adoption, abandonment). To assess this complex socio-ecological system, we developed a multistate matrix model of cats in urban areas that include owned cats, unowned cats (free-roaming and feral), and cats that move through the shelter system. Our model requires three inputs—location, number of human dwellings, and urban area—to provide testable predictions of cat abundance for any city in North America. Model-predicted population size of unowned cats in seven Canadian cities were not significantly different than published estimates (p = 0.23). Model-predicted proportions of sterile feral cats did not match observed sterile cat proportions for six USA cities (p = 0.001). Using a case study from Guelph, Ontario, Canada, we compared model-predicted to empirical estimates of cat abundance in each subpopulation and used perturbation analysis to calculate relative sensitivity of vital rates to cat abundance to demonstrate how management or mismanagement in one portion of the population could have repercussions across all portions of the network. Our study provides a general framework to consider cat population abundance in urban areas and, with refinement that includes city-specific parameter estimates and modeling, could provide a better understanding of population dynamics of cats in our communities.

## Introduction

Concerns over the population density of free-roaming domestic cats (*Felis catus*) in urban areas is a global issue centered on the issues of cat welfare [1–3], impacts on wildlife populations [4–6], and risks to public health [1, 7, 8]. Central to understanding the impacts of cats, and a first step in developing humane and cost-effective management strategies, is quantifying population abundance and identifying the factors that drive population dynamics. To address homelessness, over-taxed animal shelters, environmental impacts, public health risks, and unnecessary euthanasia we must consider the collective subpopulations of owned cats, unowned free-roaming cats, and cats in the shelter system simultaneously because each subpopulation contributes differently to the population dynamics and abundance of cats (e.g. differences in neuter rates, survival, or fecundity). In addition, cats move among subpopulations through human interventions (e.g. adoption, abandonment). Bringing together stakeholders to identify and prioritize common societal goals to address cat population densities depends on robust population abundance estimates and understanding how interventions will reverberate across the population network.

National population estimates suggest about 100 million owned cats (indoor and indoor/outdoor; [1, 3, 6]) and between 10 and 120 million unowned (free-ranging and feral; [1, 5, 6]) live in the United States and Canada. In urban areas, the debate around cat management centers on the use and effectiveness of lethal and non-lethal control measures to address concerns over the free-roaming cat population density [1, 9, 10]. While the objectives of such control measures are rarely stated explicitly, owing to the variety of stakeholder views on the role, rights, and impact of cats, generally public expectations relate to issues of ethical population control, the cost and effectiveness of implementing these controls, and the likelihood of achieving a specified management objective [11, 12].

A number of population models for cats have been developed to understand population dynamics and to quantify how interventions are likely to reduce population abundance. To date, most population modeling efforts have focused on feral cats [13, 14], considered a limited set of interventions focused on lethal culls or sterilization [14–20], and largely ignored the transition of cats between subpopulations except for low rates of abandonment [14, 19, 20]. While these models are useful, it is important to recognize that they have focused on a limited portion of the total cat population and have considered the characteristics of cat population sizes specific to very small urban areas [21]. Therefore, previous models are not necessarily suitable for understanding the dynamics of high population densities such as those found in large urban areas [22, 23]. To address these limitations, a population model that accurately predicts population abundance, can identify the factors that are driving population dynamics of cats and requires easily available inputs (e.g. from human census data) for any urban area is necessary. Such a population model could provide information to decision-makers that must choose among several competing and emotionally charged interventions that target demographic vital rates in different subpopulations, to influence cat populations [10, 24, 25].

Our objectives were to (i) develop a multistate matrix population model for owned cats, unowned cats, and cats that move between these two categories via the shelter system applicable to any city in North America using simple inputs available in national census data sets, (ii) assess model fit by comparing model-derived population abundance predictions of urban areas where cats, and unowned cats especially, have been estimated, and (iii) determine the sensitivities of population abundance to demographic vital rates and transition across the entire cat population network for Guelph, Ontario, Canada where previous cat population estimates [23, 26] and shelter statistics [27] are available.

## Methods

**Fig 1:**
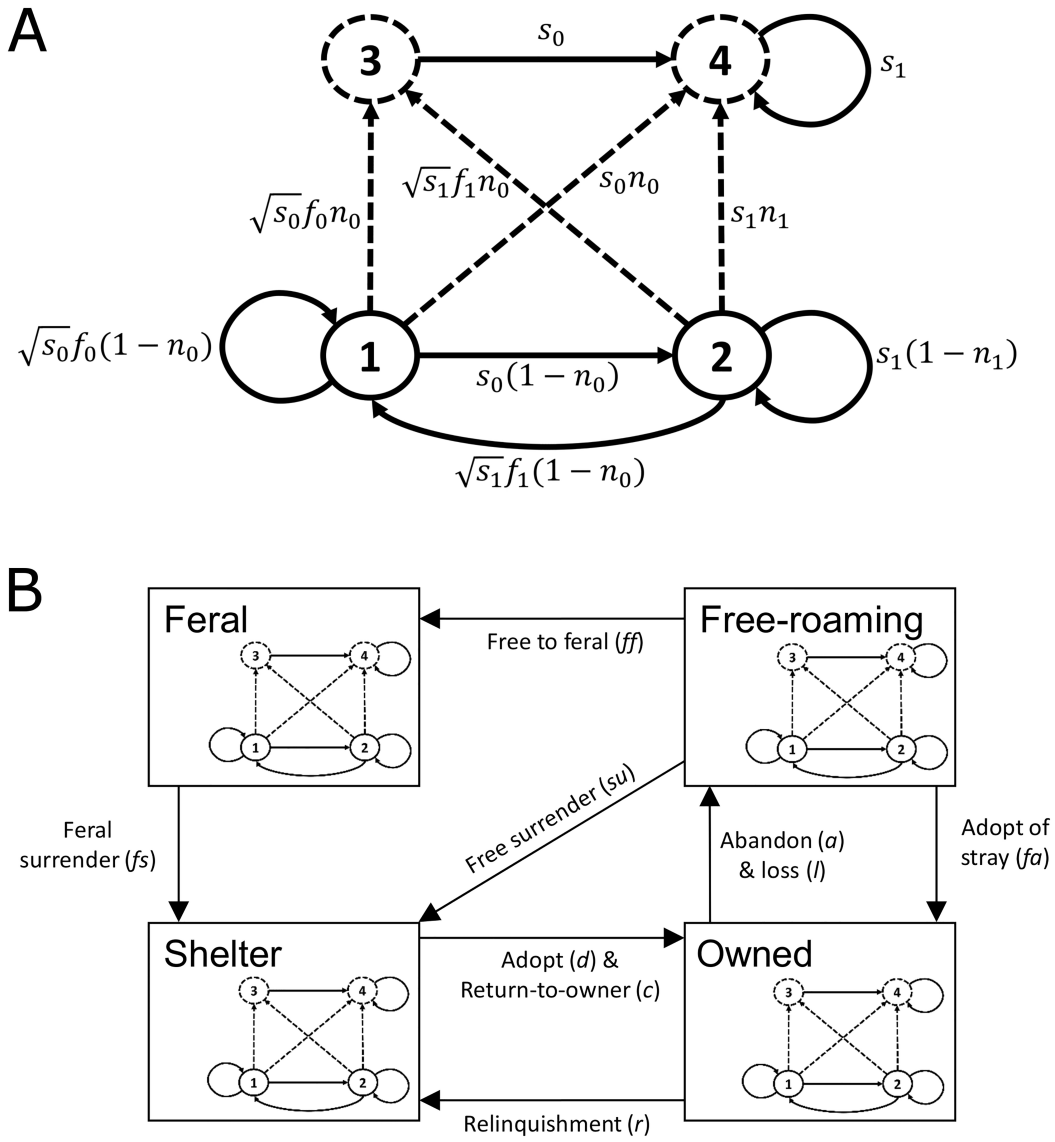
A schematic diagram of the population model for cats. (A) Biological life-cycle diagram for domestic cats. Stage 1 are intact juvenile cats, stage 2 are intact adult cats, stage 3 are sterilized juvenile cats, and stage 4 are sterilized adult cats. Parameters shown include survival (*s*), fecundity (*f*), and sterilization *(n*); the values are presented in Table 1. Solid circles indicate intact life stages (reproductive) while dashed circles indicate sterilized life stages (non-reproductive) age categories. The time of sterilization within the life cycle is indicated with dashed lines. Subscripts on vital rates refer to kittens (0) and adults (1). (B) State and transition of domestic cats. Models for cities in North America considered in this paper used a four-state model that classified unowned cats as either free-roaming or feral cats. Note that the same biological life-cycle graph in (A) is found in each state in the model but will have different values for demographic vital rates.

We used a life stage- (*s* = 4) and state-structured (*r* = 4) birth-pulse discrete periodic matrix model, with density dependence attributes, using the vec-permutation approach [28, 29]. Here, *s* refers to the life stage of an individual based on age (juvenile, adult) and reproductive stage (intact, sterile): stage 1 are intact kittens, stage 2 are intact adults, stage 3 are sterilized kittens, and stage 4 are sterilized adults (Fig 1A). While there are multiple ways to sterilize cats that may influence population dynamics through cat social structure at the individual level [18], we consider no individual social effects with sterilization akin to neutering cats when considering population-level processes. At each time step, deemed here to be 1-year, *r* considers cats as being in one of four states: state 1 are owned cats, state 2 are cats in the shelter system, state 3 are free-roaming cats, and state 4 are feral cats (Fig 1B). State 3 and 4 are collectively unowned cats but free-roaming cats comprised stray, abandoned, or lost cats that had been previously socialized to people and therefore were candidates to be surrendered to an animal shelter system or adopted by citizens off the street whereas strict feral cats are neither socialized to people nor candidates to be adopted (Fig 1B). Matrix models only consider females under the assumption that males are not limiting [30] but for model fit we assumed a 1:1 sex ratio to compare our population predictions to published estimates of cat abundance. Although cat population density is stochastic based on a variety of environmental [31] and social [32] factors, we have assumed the model to be deterministic recognizing that this currently limits the applicability of the results of our model analysis.

**Table 1:** Demographic vital rates used in a multistate matrix population model of domestic cats with an annual time step. Presented are survival (*s*), sterilization rate (*n*), and fecundity (*f*) for owned cats, free-roaming cats, feral cats, and cats moving between these states via the shelter system. Survival and sterilization are annual probabilities whereas fecundity is the number of female kittens produced per female per year. Some vital rates are density dependent. Listed are the state and stage of the cat, the variable in the population model, the value or equation for the vital rate, and references that inform parameterization. Note that values may differ slightly from those presented in the original source and that if left blank the variable was assumed for this model. The number of litters per female per year was k=y149 where 149 is the number of days required to produce 1 litter (pregnancy lasts 65 days, weaning young requires 84 days) and *b* is the length of the breeding season in days.

State	Stage	Variable In Eq (7)	Value or equation	Reference
*Survival (s)*				
Owned	Juvenile	s_0_	0.7	[33]
Owned	Adult	s_1_	0.8	[33]
Shelter	Juvenile	s_0_	0.7 × (1 − 0.519)	[3, 33]
Shelter	Adult	s_1_	0.8 × (1 − 0.519)	[3, 33]
Free-roaming	Juvenile	s_0_	$\left(-0.5867\frac{N_{free\left(Intact\;adults\right)}}{N_{free\left(Sterile\;adults\right)}+N_{free\left(Intact\;adults\right)}}+0.9067\right)\times \sqrt{0.82e^{-0.475669\frac{N_{f}}{city\;area}}}$	[13, 34]
Free-roaming	Adult	s_1_	$\sqrt{0.82e^{-0.475669\frac{N_{f}}{city\;area}}}$	[35–37]
Feral	Juvenile	s_0_	$0.25e^{-10.0719\frac{N_{feral}}{city\;area}}\times \sqrt{0.65e^{-10.0719\frac{N_{feral}}{city\;area}}}$	[13, 38]
Feral	Adult	s_1_	$0.65e^{-10.0719\frac{N_{feral}}{city\;area}}$	[35, 39]
*Fecundity (f)*				
Owned	Juvenile	f_0_	$0.4192\frac{3.8k}{2}$†*	[13, 40, 41]
Owned	Adult	f_1_	$\frac{3.8k}{2}$†*	[40, 41]
Shelter	Juvenile	f_0_	0	
Shelter	Adult	f_1_	0	
Free-roaming	Juvenile	f_0_	$0.4192\frac{3.6k}{2}$†*	[13, 35, 41]
Free-roaming	Adult	f_1_	$\frac{3.6k}{2}$†*	[35, 41]
Feral	Juvenile	f_0_	$0.4192\frac{3.5k}{2}$†*	[13, 35, 41]
Feral	Adult	f_1_	$\frac{3.5k}{2}$†*	[35, 41]
*Sterilization (n)*				
Owned	Juvenile	n_0_	0.7	[33]
Owned	Adult	n_1_	0.1	[33]
Shelter	Juvenile	n_0_	1	
Shelter	Adult	n_1_	1	
Free-roaming	Juvenile	n_0_	0.05	[42]
Free-roaming	Adult	n_1_	0.125	[42]
Feral	Juvenile	n_0_	0.01	[42]
Feral	Adult	n_1_	0.025	[42]

* assumes 1:1 sex ratio

^†^ intact cats

Periodic models allow concurrent consideration of demographic vital rates (survival, fecundity, and sterilization) of individuals within each stage followed by transition of individuals (e.g. adoption, relinquishment, abandonment, loss) amongst the different states of the urban cat population (Fig 1B; [24, 30]). The population is described by the matrix

$$N\left(t\right)=\left(\begin{array}{ccc} n_{11} & \cdots & n_{1r}\\ \vdots & \ddots & \vdots \\ n_{s1} & \cdots & n_{sr} \end{array}\right)\left(t\right)$$ (1)

$$\text{vec}N^{T}\left(t\right)=n\left(t\right)=\left(\begin{array}{c} \begin{array}{c} n_{11}\\ \vdots \\ \underline{n_{1r}} \end{array}\\ \vdots \\ \begin{array}{c} \bar{n_{s1}}\\ \vdots \\ n_{sr} \end{array} \end{array}\right)\left(t\right)$$ (2)

where the entries of the population are transformed into a vector **n** at time *t* ordered by stages and then by states so the first entry *n*_11_ is stage = 1, state = 1, the second entry *n*_12_ is stage = 1, state = 2, etc. [28, 29]. The matrix model took the form

n(t+1)=An(t) (3)

$$A=MKBK^{T}$$ (4)

where **A** projects the population vector **n** from time t to t+1. From right to left, **K**^*T*^ is the transpose vec-permutation matrix which rearranges the population vector such that the matrix B accounts for demography within states, **K** then returns the population vector to its original form where the matrix M moves individuals between states [28, 29, 43]. In this arrangement, annual demographic processes occur before transitions among states. The demography of individuals in state *i* is given by an *s* × *s* projection matrix **B**_*i*_ while transition of individuals in stage *j* is an *r* × *r* matrix **M**_*j*_ contained within the block-diagonal matrices B and M, respectively:

$$B=\left(\begin{array}{ccc} B_{1} & \cdots & 0\\ \vdots & \ddots & \vdots \\ 0 & \cdots & B_{r} \end{array}\right)$$ (5)

$$M=\left(\begin{array}{ccc} M_{1} & \cdots & 0\\ \vdots & \ddots & \vdots \\ 0 & \cdots & M_{s} \end{array}\right)$$ (6)

Population dynamics are thus described in terms of exchanges between demography among stages and transition among states.

Within each state (subpopulation) of the population, **B**_*i*_, cats were considered as juvenile (<12 months of age) or adult (>12 months of age) and as reproductively intact or sterilized (Fig 1A):

$$B_{i}=\left[\begin{array}{cccc} \sqrt{s_{0}}f_{0}\left(1-n_{0}\right) & \sqrt{s_{1}}f_{1}\left(1-n_{1}\right) & 0 & 0\\ s_{0}\left(1-n_{0}\right) & s_{1}\left(1-n_{1}\right) & 0 & 0\\ \sqrt{s_{0}}f_{0}n_{0} & \sqrt{s_{1}}f_{1}n_{1} & 0 & 0\\ s_{0}n_{0} & s_{1}n_{1} & s_{0} & s_{1} \end{array}\right]$$ (7)

where *s*_0_ and *s*_1_ are the annual survival of juvenile and adult respectively, *n* is the probability of sterilization, and *f* is fecundity expressed as the number of female kittens per female per year following similar notation for stage structure in all vital rates. The first two columns represent intact individuals while the last two columns represent sterilized individuals (Fig 1A). Including a sterile life stage is analogous to incorporating a post-reproductive life stage in a projection matrix model (e.g. [44]). Incorporating these details is important to track changes in stage densities that can be compared to observed data on population structure (i.e. proportion of the population that is comprised of post-reproductive individuals) yet allows the model to account for estimating the effective population size which involves only reproducing individuals [30, 45].

For each stage (e.g. intact kittens) of the population, **M**_j_ (Fig 1B), the transition of cats among being owned, free-roaming, feral, or in the shelter system is:

$$M_{j}=\left[\begin{array}{cccc} 1-(r_{1}+l_{1}+a_{1}) & d_{1}+c_{1} & {fa}_{0} & 0\\ r_{1} & 1-(d_{1}+c_{1}) & {su}_{0} & {fs}_{1}\\ l_{1}{\;+\;a}_{1} & 0 & 1-({fa}_{1}+{su}_{1}+{ff}_{1}) & 0\\ 0 & 0 & {ff}_{1} & 1-({fs}_{1}) \end{array}\right]$$ (8)

where *r* is relinquishment (surrendering an owned cat to an animal shelter), *l* is loss, *a* is abandonment (the intentional release of an owned cat outdoors thereby making it unowned), *d* is adoption, *c* is return-to-owner of a lost cat, *fs* is surrender of feral, *fa* is adoption of free-roaming cat, *ff* are kittens of free-roaming cats that become feral due to lack of exposure to humans, and *su* is surrender of free-roaming cat to an animal shelter. These data present the proportion of the population that move between states dependent upon survival from the source subpopulation and thus assume no mortality during transition.

### Parameter values

The model requires a large number of parameters that includes both demographic vital rates and transition probabilities. Several parameter values are estimated across a number of studies and sample sizes in most of these studies are small that made generalizing parameter inputs challenging. In some cases, there are no parameter estimates available, especially for the state transition probabilities. In cases such as these in the ecological literature, educated guesses of the strength and form of relationships for plausible parameter estimates based on the expected behaviour of the system dynamics and allometric reasoning is often used [46–48]. Sensitivity analysis (see below) provides a means to retrospectively determine which of these parameters (if incorrectly specified), has the greatest impact on model outcomes [43, 49].

### Carrying capacity

As our model was deterministic, we estimated carrying capacity for each state to enforce population regulation that resulted in a steady-state. The concept of carrying capacity has been largely ignored for cats in the literature so our estimates are approximations based on the assumption that empirical cat population density measurements reflect populations at equilibrium (e.g. [21–23]). In the model, carrying capacity of each subpopulation was dependent upon a variety of factors such as urban area size and latitude (the latter influences variation in reproductive output, see below) for unowned cats. We used the carrying capacity to then derive density-dependent functions of survival within states and transition rates among states.

#### Owned cats

Carrying capacity for owned cats (*K*_*o*_) was determined using a standard equation for the number of dwellings (*h*) multiplied by the proportion of dwellings with cats multiplied by the number of cats in occupied dwellings [3, 50].

$$K_{o}=\left(0.377\times 1.85\times h\right)/2$$ (9)

The proportion of dwellings with cats came from standardized polling data from Canada which found 37.7% of dwellings owned a cat and that each of these dwellings had, on average, 1.85 cats [3]. Because our assumption is an equal sex ratio, dividing the numerator by 2 results in the estimated carrying capacity of owned female cats in an urban area. These values for Canada are generally higher than equivalent data published in the United States of 21% and 1.66 cats/dwelling [51], 19–26% and 1.43–1.66 cats/dwelling in the UK [50, 52], 10.4% and 1.6 cats/dwelling in Ireland [53], and 15% and 1.7 cats/dwelling in Italy [54]. To estimate the number of dwellings in an urban area (*h*) for the cities considered for model validation purposes (see below), we used geo-referenced census data for Canada (‘population centres’ which are urban areas with a population size >1000; [55, 56]) and the United States (‘urban areas’ which encompass residential, commercial, and other non-residential urban lands and have a population size >2500; [57, 58]) or information reported on the homepage of focal cities in North America.

#### Shelter cats

The carrying capacity for shelters (*K*_*s*_) is analogous to annual shelter capacity that is commonly considered in animal shelter welfare studies [59]. We determined the carrying capacity for shelters using two variables. The first was the number of spaces for cats, generally termed the capacity (*c*), of the shelter [59]. The second was dividing 365 days by the average length of stay (*s*), in number of days, for each cat. The product of this equation was the estimated maximum number of cats, per year, that the shelter can accommodate.

$$K_{s}=\frac{365c}{s}$$ (10)

The capacity of shelters was derived from the mean value of reporting humane societies in Canada from [3] which was a capacity of 187 cats. It should be noted that capacity of shelters is likely dependent upon the urban area extent (i.e. the number of households to be served), the number of other shelters or similar organizations within the urban area, human population density, and socio-economic factors [59]. The prediction is that larger shelter capacities occur in large urban areas with few other shelter organizations. The average length of stay was assumed to be 30 days which is between the mean (49 days) and median (27 days) length of stay for cats based on a survey performed in Canada [60].

The value *K*_*s*_ could represent a carrying capacity ceiling of the shelter, in the sense that if cats enter the shelter beyond this value, then the excess would be euthanized or released. While we were strictly interested in stable-states, this formulation could be used in conjunction with relaxing density-dependent transition rates to model the effects of proposed no-kill policies or limited/closed admission approaches for dealing with stray cats.

#### Unowned cats

There are few estimates of carrying capacity for unowned cats. To derive carrying capacity estimates of free-roaming and feral cats we compiled published estimates of cat density (cats/ha), or the number of cats and associated study area (ha), and used mean values of these estimates to derive a city-specific carrying capacity for free-roaming and feral cats. Density estimates for free-roaming cats came from studies conducted in urban areas, cat colonies, and general studies on mainland areas where cats were associated with humans or cared for by humans in outdoor settings (S1 Table). In contrast, density estimates for feral cats came largely from studies of island populations (S1 Table). Taking this approach, feral cats occur at lower density than free-roaming cats.

**Fig 2:**
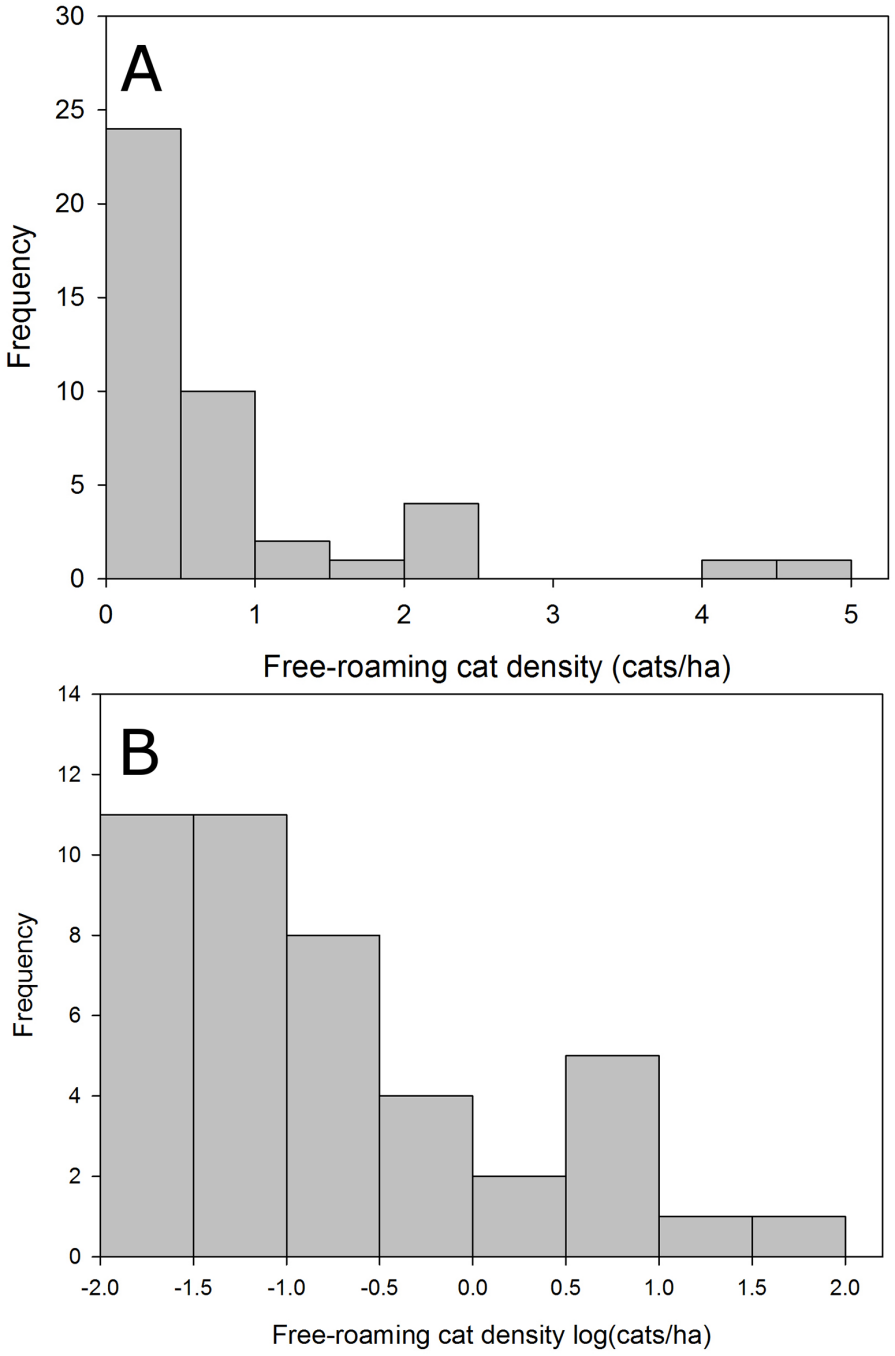
Published estimates of free-roaming cat density. Histogram of (a) population density and (b) log-transformed population density estimates for free-roaming cats used to estimate carrying capacity. Mean density (*e*^log(density)^ = 0.4711098 cats/ha) was calculated using the log-transformed estimates of free-roaming cat density from the literature. For our female-only models, we considered an equal sex ratio and so these values presented here were multiplied by 0.5 to get estimates of female free-roaming cat densities.

**Fig 3:**
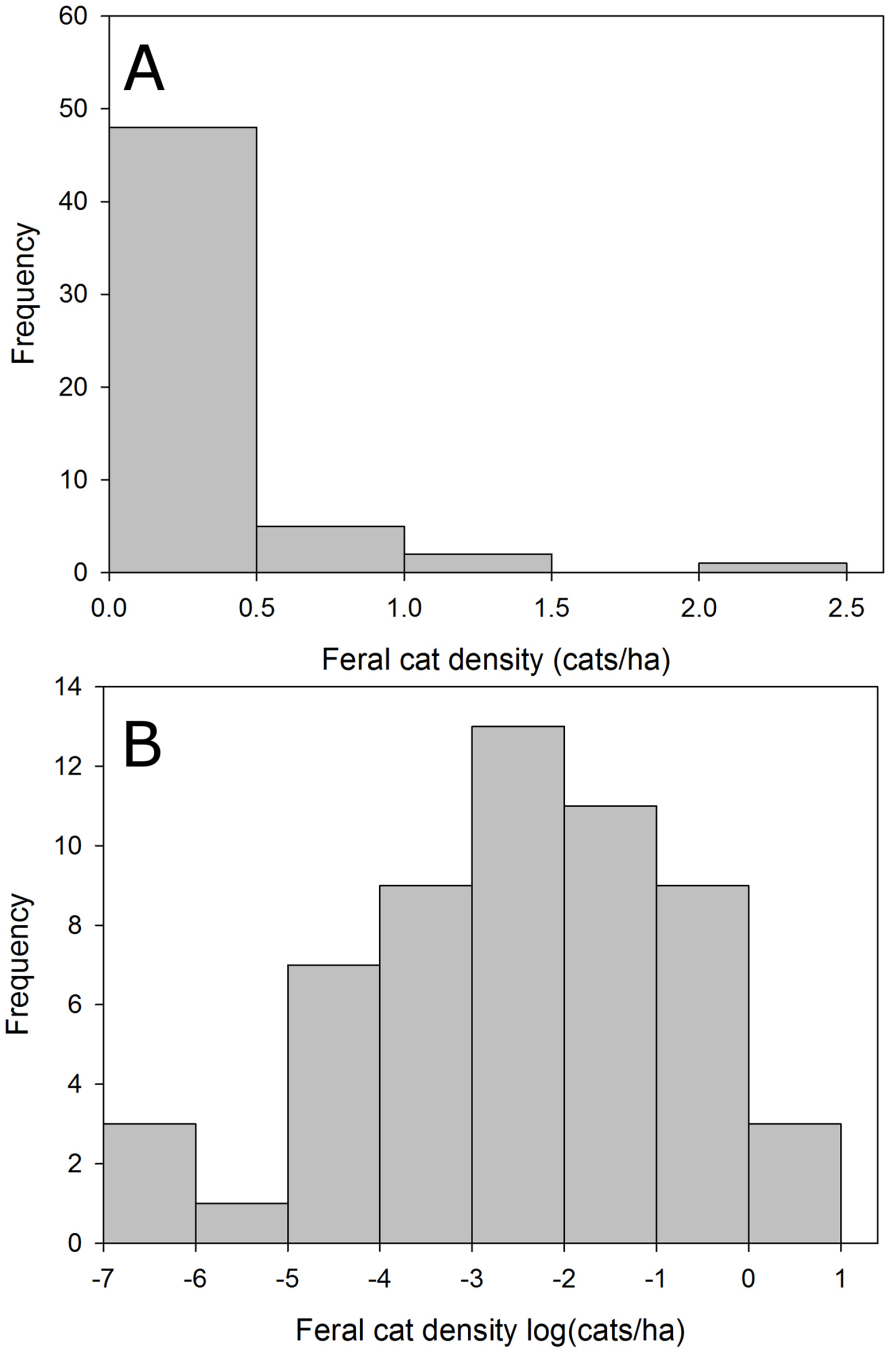
Published estimates of feral cat density. Histogram of (a) population density and (b) log-transformed population density estimates for feral cats used to estimate carrying capacity. Mean density (*e*^log(density)^ = 0.07911264 cats/ha) was calculated using the log-transformed estimates of feral cat density from the literature. For our female-only models, we considered an equal sex ratio and so these values presented here were multiplied by 0.5 to get estimates of female feral cat densities.

Raw data for both free-roaming (range: 0.15–4.88 cats/ha) and feral cats (range: 0.001–2.43 cats/ha) were positively skewed (Figs 2A and 3A), so we log-transformed the cat density data and then used the mean value in an exponential function to estimate the deterministic cat density at carrying capacity for free-roaming (*e*^log(density)^ = *e*^-0.752664^ = 0.4711098 cats/ha) and feral cats (*e*^log(density)^ = *e*^-2.536883^ = 0.07911264 cats/ha; S1 Table). Because the log-transformed data follows an approximate normal distribution (Figs 2B and 3B), these data could be suitable for stochastic simulations. Urban land area was obtained from a geographic information system that provided urban extents for all cities in Canada and the United States [56, 58]. Multiplying mean free-roaming or feral cat density by urban land area extent provides an estimate of the carrying capacity for each unowned cat population.

### Reproduction

#### Fecundity

Per capita reproductive output is the product of the number of female kittens per litter and the number of litters per year. Cats are seasonal breeders that can produce multiple litters per year [38, 61]. In temperate environments, seasonality is the duration of the reproduction period driven predominately by photoperiod [41, 62]. Modeling breeding season length with respect to the latitude of the urban area and the gestation period of cats therefore seems a reasonable way to estimate the maximum number of litters that could be produced by each intact female cat per year.

**Fig 4:**
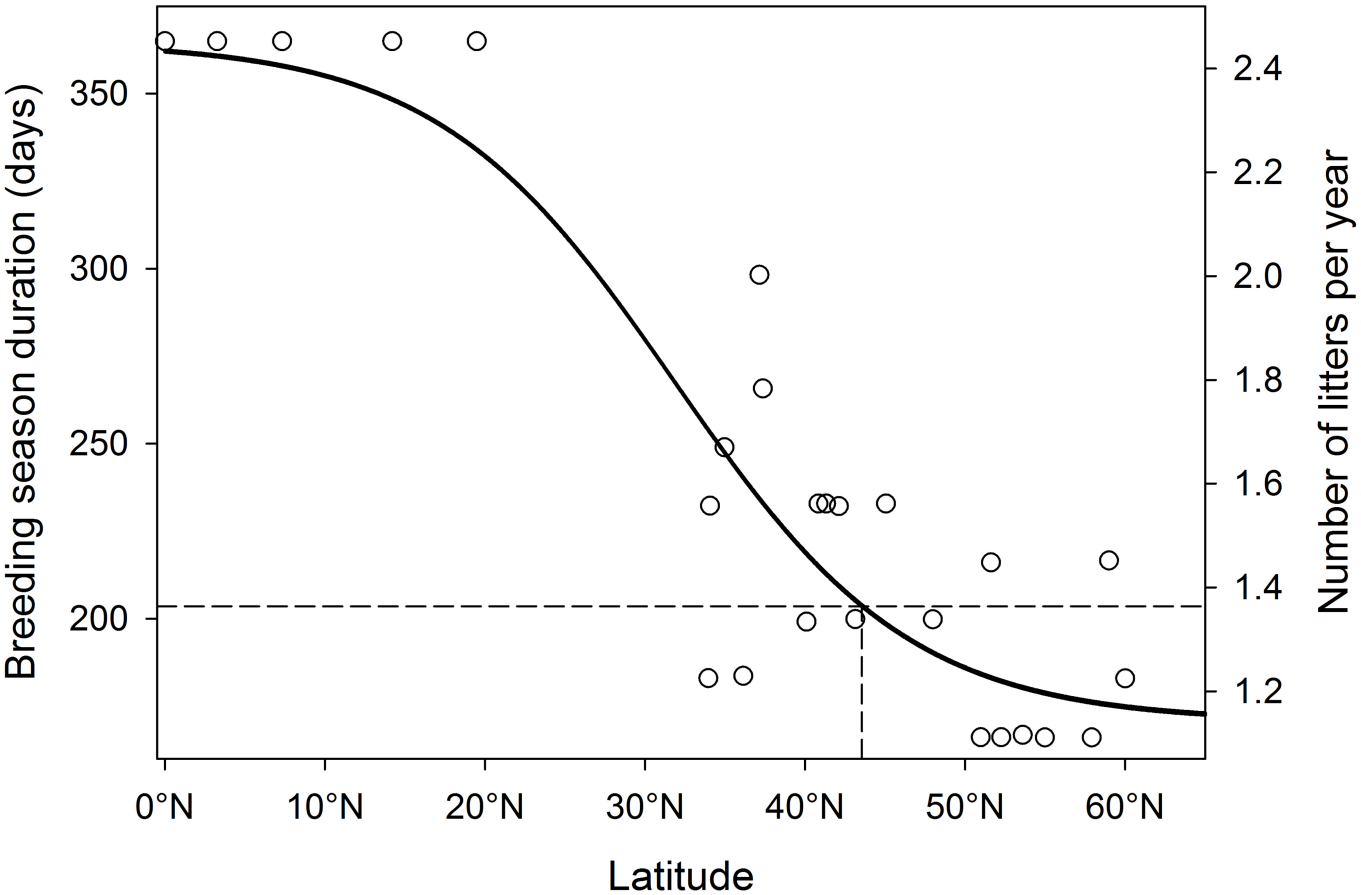
Geographic variation in breeding season duration and the number of litters per year for cats. Breeding season duration (left axis) given latitude and the corresponding average number of litters per female per year (right axis). The data used to fit the model are the dots and are from Hurni [41]. The line is the 4-parameter logistic function and the parameter estimates are provided in Table 2. The dashed lines are the latitudes, predicted breeding season duration and mean number of litters per female per year of the case-study city of Guelph, Ontario.

The number of litters per female per year was calculated as

k=y149 (11)

where 149 is the number of days required to produce 1 litter (gestation period was estimated as 65 days [63, 64], weaning young was assumed 84 days) and *y* is the length of the breeding season in days from a logistic regression (Fig 4). We extracted the data from [41] and fit a nonlinear, 4-parameter logistic regression model of the breeding season length *y*, in days, given a latitudinal coordinate. The logistic function took the form:

$$y=a+\frac{b-a}{1+e^{c(d-x)}}$$ (12)

where *x* is latitude and the other terms are estimated parameters from the regression model (Table 2). Parameter *a* is the y-intercept when *x* is 0, and we fixed this parameter to 365 to ensure that the maximum breeding season length was year-round [41]. The estimate of *k* in Eq (11) ignores the amount of time it takes females to become pregnant but does capture the time delay from failed pregnancies (i.e. pseudopregnancy) in the estimates of breeding season length. For the focal cities, we derived latitude at the center of the urban extents from the geographic information system for cities in Canada and the United States. Our modeled relationship of *k* was suitable when compared to the number of litters from field studies, for example, Nutter et al. [38] measured 1.4 litters per year per female in North Carolina (latitude 37.4N) which aligned well with our estimated relationship (Fig 4).

**Table 2:** Parameter estimates for a 4-term logistic function of breeding season length of cats $y=a+\frac{b-a}{1+e^{c(d-x)}}$ where *x* is latitude. The parameter *a* was fixed at 365 to ensure that at low latitudes that the breeding season duration did not exceed 365 days. Data to estimate the logistic function were derived from Hurdi [41] and both the data and the function are presented in Fig 4.

Parameter	Estimate	SE	t-value	P-value
A	365 (fixed)	-	-	-
B	170.3539	15.7057	10.847	<0.001
C	0.1351	0.0483	2.798	0.001
D	31.8447	2.3768	13.398	<0.001

The number of female kittens per litter was estimated for adults (> 1 year) and kittens reproducing in their first year (<1 year) in each category. Owned adult females produced 1.9 female kittens [40], free-roaming adults produced 1.8 female kittens [35], and feral adults produced 1.75 female kittens [35]. The number of offspring that kittens produce in their first year is reduced compared to adults because most are pre-pubertal. We used a mean pubescent date of 212 days to calculate the proportion of kittens that would breed in their first year ((365–212)/365; [13]) and multiplied this by the reproductive output of adults in each category. For both age categories, we assumed cats in the shelter system were prevented from breeding. Finally, we assumed a 1:1 sex ratio.

#### Sterilization rate

The annual proportion of the population that produces offspring is calculated by subtracting the proportion of the population that is annually sterilized from one (Eq (7)). However, the proportion of cats that are sterilized varies substantially between different subpopulations of cats. Although studies have found that socio-economic factors influence the probability of sterilizing an owned pet [65], we assumed a simple relationship that 70% of kittens were sterilized in their first year and thereafter 10% of remaining intact adults were sterilized annually by their owners [33]. Shelters may not sterilize all cats before they are adopted [3] but we assumed that all cats moving through the shelter system were sterilized before being placed into homes. The annual sterilization probability of free-roaming and feral cats was based upon the proportion of cats submitted to trap-neuter-return programs that are already sterilized. The proportion of the population already sterilized ranged from 0.7% to 3.5% [42] so we assumed 1% of kitten and 2.5% of adult free-roaming cats are annually sterilized and 0.1% of kitten and 0.25% of adult feral cats are sterilized through trap-neuter-return programs. We assumed this difference between free-roaming and feral cats based on disparity between detectability and trapability of these two groups of cats.

### Survival

The probability of annual survival of cats was considered as either density-dependent or density-independent. Survival of owned cats was considered density-independent as we reasoned that owned cats neither compete nor are limited by resource availability such as food. Annual survival of kittens was assumed to be 0.7 and for adults was 0.8 which is below estimates in the literature from an urban area in Manhattan, Kansas which were 0.8 for juveniles in their first year and 0.9 for adult cats [33]. We assumed that survival was the same for intact and sterilized owned cats.

Survival of cats in the shelter system was considered density-independent, however, cats in shelters are at increased risk of euthanasia. Therefore, we considered survival to be the same as owned cats of each age, however, we also removed a proportion of cats from the population that we considered would be removed via euthanasia. Shelter questionnaire data from Canada reported by the Canadian Federation of Humane Societies [3] indicated that 39.9% of cats are euthanized and an additional 12% were without a home. Assuming cats without a home are at risk of euthanasia, then 51.9% of cats that enter shelters are likely euthanized while 48.1% of shelter cats will survive. These values are in general agreement with shelter statistics reported in the USA (65% of cats were euthanized at 186 shelters and animal control agencies across 42 states; [59]). We assumed that survival was the same for intact and sterilized owned cats.

**Fig 5:**
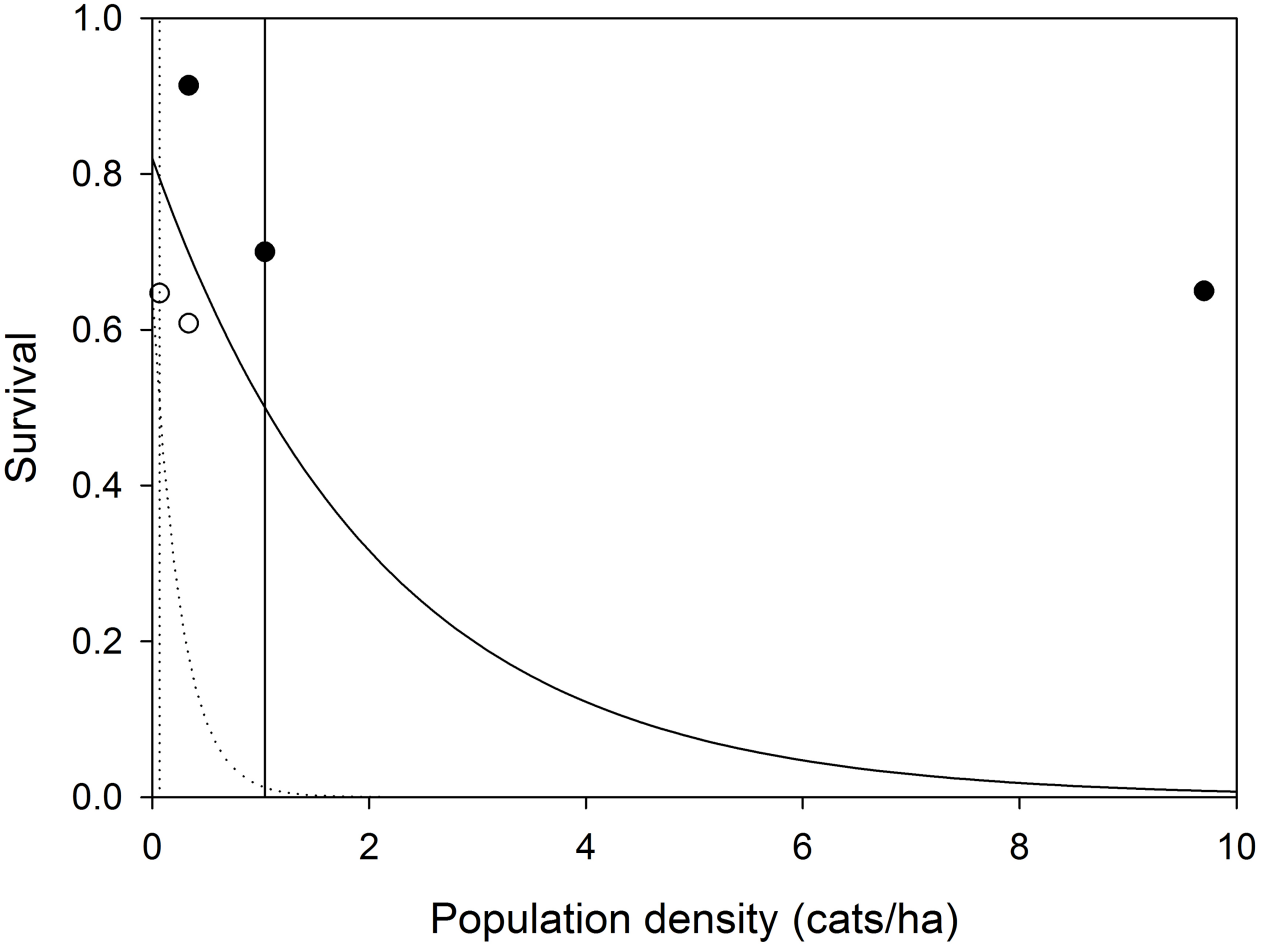
Density dependent survival of adult free-roaming (solid) and feral (dashed) cats. Vertical lines are the estimated carrying capacity for each group. Points (solid, free-roaming; hollow, feral) are observed estimates of survival for free-roaming [35–37] and feral cats [35, 39].

Survival of unowned cats was considered to be density-dependent with respect to the carrying capacity of free-roaming and feral cats (Fig 5). The carrying capacity, and hence strength of density-dependent survival, of unowned cats is thought to be dependent upon food availability [66]. We plotted estimated local survival probability and density [35, 36] or, where density was not reported by the investigators [37, 39], we assumed it was at the mean carrying capacity, and fit exponential decay curves of the form

$$y=ae^{\left(-b\;\times Density\right)}$$ (13)

to represent the density-dependent survival rate. Because data were limited, we reiterated these functions until they met the assumption of annual adult survival probability of 0.5 at the mean carrying capacity which was *e*^-0.752664^ (0.4711098 cats/ha) for free-roaming cats and *e*^-2.536883^ (0.07911264 cats/ha; S1 Table) for feral cats, as described above. For annual kitten survival, we used the product of the lower survival probability during the first 6-months of life and the next 6-months was estimated at the adult survival rate (i.e. the square root of annual adult survival) [13].

**Fig 6:**
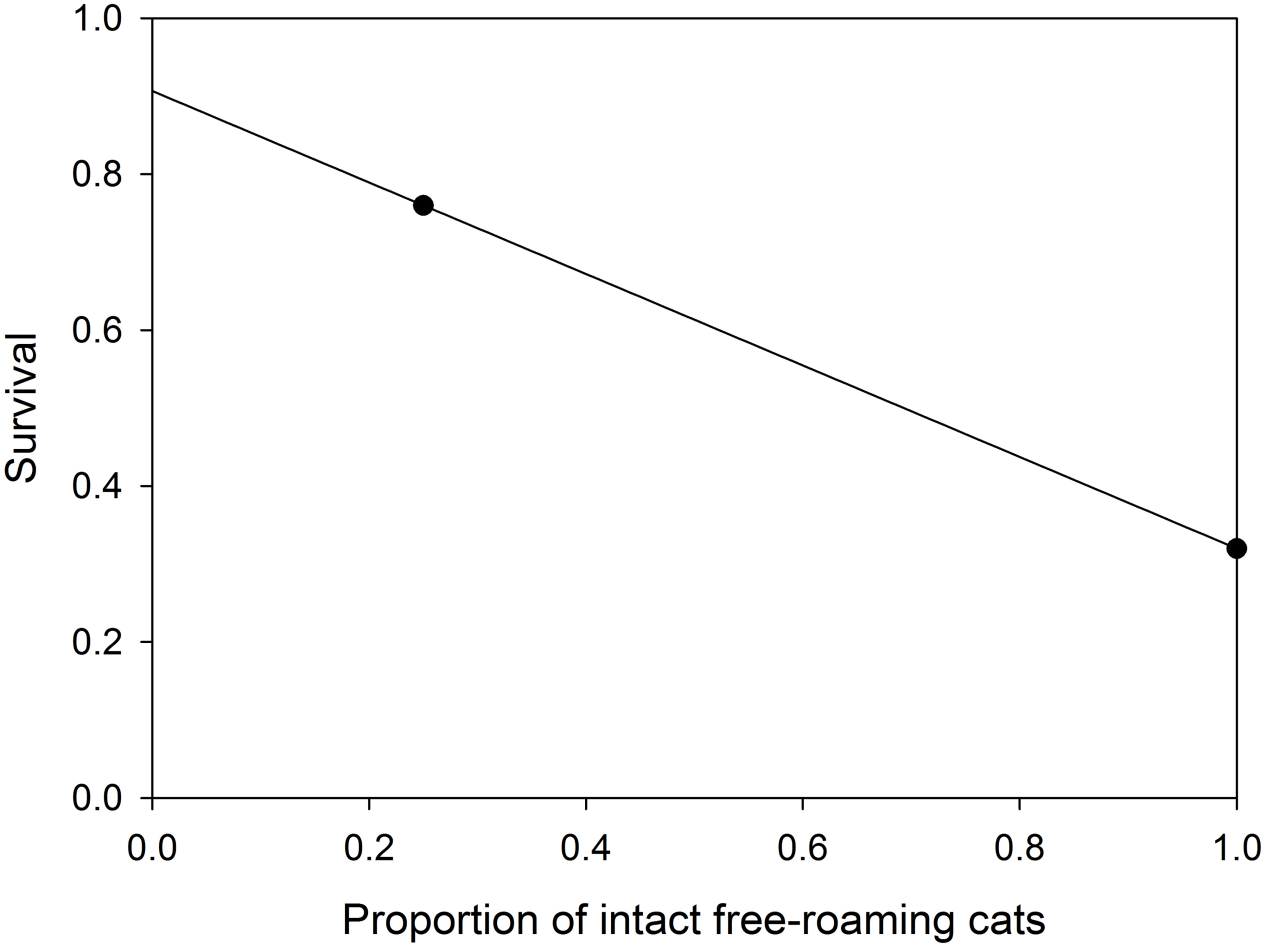
Survival of juvenile free-roaming cats. Frequency-dependent survival of juvenile free-roaming cats in the first 6 months of life and the proportion of the adult population that is intact. The points are those observed by Gunther et al. [34] in two colonies in Israel with different proportions of intact adult cats.

For adult free-roaming cats, we used three studies [35–37] that reported annual survival that ranged from 0.65–0.91 and density that ranged from 0.34–9.7 cats/ha (Fig 5). The fitted relationship (Fig 5) was

$$0.82e^{-0.476e^{0.0482}(N/K)}$$ (14)

where K is the city-specific carrying capacity based on urban area extent as described above and N is the population abundance of free-roaming cats. For kittens, there is support for the hypothesis that hormone-mediated aggressive interactions result in higher mortality in fully intact populations [34] which has implications for the effectiveness of interventions aimed at reducing population size [14, 18]. Therefore, we considered density-dependent survival to vary by the proportion of the population that is sterilized in free-roaming kittens based on the data presented in Gunther et al. ([34]; Fig 6). We regressed the proportion of intact cats in two free-roaming cat colonies against the survival probability estimates of juveniles at 6-month of age in these colonies presented in Gunther et al. [34] to fit the linear relationship

$$-0.587(\frac{N_{intact}}{N_{total}})+0.907$$ (15)

where *N* refers only to adult free-roaming cats (Fig 6). The frequency-dependent component of this survival assumes that cats occur at high densities and are subject to aggressive interactions from conspecifics, which we reason could occur in cat colonies supplemented by humans.

For adult feral cats, we used two studies [35, 39] whose annual survival ranged from 0.61–0.65 and density ranged from 0.068–0.34 (Fig 5). The fitted relationship (Fig 5) was

$$0.65e^{-3.812e^{-2.676}(N/K)}$$ (16)

as above except that N and K pertain to the abundance and carrying capacity of feral cats (Fig 5). For feral kittens, we applied the intercept of 0.25 following Nutter et al. [38] and continued with the slope of the adult survival rate under density dependence. The form of the equation for density-dependent survival of feral kittens was therefore

$$0.25e^{-3.812e^{-2.676}(N/K)}.$$ (17)

### Transition probabilities

Transition probabilities are the annual proportion of a subpopulation that moves among states (Fig 1A). Note that the transition is for each stage (age/intact status) class and hence specific applications could account for differential transition probabilities based on age, sex, or reproductive status; this is commonly reported in studies looking at owner preferences in adoptions (e.g. [67]). We consider that transitions are consistent among all stages except for kittens of free-roaming cats that become feral and for differential return-to-owner probabilities between intact and sterilized owned cats (see below). Values and equations for transition probabilities are provided in Table 3.

**Table 3:** Transition among states in a multistate matrix population model of domestic cats with an annual time step. Listed are the variable, transition between states, the equation for the vital rate, the value, and references that inform parameterization. Note that some values may differ slightly from those presented in the original source and that if left blank the variable was designated specifically for this model.

Variable	Transition	Variable In Eq (8)	Value	Reference
Remain owned	Owned to Owned	1 –(r + l + a)	0.9331	calculated
Relinquishment	Owned to Shelter	r	0.012	[68]
Lost	Owned to Free-roaming	l	0.0049	[69, 70]
Abandonment	Owned to Free-roaming	a	0.05	[71]
Remain Shelter	Shelter to Shelter	1 –(d + c)	$1-(\frac{e^{-12\frac{N_{o}}{K_{o}}+12}}{1+e^{-12\frac{N_{o}}{K_{o}}+12}}-0.03847807)$^†^ $1-(\frac{e^{-12\frac{N_{o}}{K_{o}}+12}}{1+e^{-12\frac{N_{o}}{K_{o}}+12}}-0.08773)$^††^	calculated
Adoption	Shelter to Owned	d	$\frac{e^{-12\frac{N_{o}}{K_{o}}+12}}{1+e^{-12\frac{N_{o}}{K_{o}}+12}}$	[3]
Return-to-owner	Shelter to Owned	c	0.03847807^†^ 0.08773^††^	[70, 72]
Remain Free-roaming	Free-roam to Free-roam	1 –(fa + su + ff)	$1-\left(\left[e^{-1.20397\left(\frac{N_{o}}{K_{o}}\right)}\;\times \;\frac{e^{3.87637\left(\frac{N_{f}}{K_{f}}\right)-5}}{1+e^{3.87637\left(\frac{N_{f}}{K_{f}}\right)-5}}\right]+\left[\frac{e^{0.1671\left(\frac{N_{f}}{K_{f}}\right)-0.5858}}{1+e^{0.1671\left(\frac{N_{f}}{K_{f}}\right)-0.5858}}\times \frac{e^{3.87637\left(\frac{N_{f}}{K_{f}}\right)-5}}{1+e^{3.87637\left(\frac{N_{f}}{K_{f}}\right)-5}}\right]+0.05\right)$	calculated
Adoption of stray off street	Free-roam to Owned	fa	$e^{-1.20397(\frac{N_{o}}{K_{o}})}\;\times \;\frac{e^{3.87637(\frac{N_{f}}{K_{f}})-5}}{1+e^{3.87637(\frac{N_{f}}{K_{f}})-5}}$	[68]
Surrender of free-roaming cat	Free-roam to Shelter	su	$\frac{e^{0.1671(\frac{N_{f}}{K_{f}})-0.5858}}{1+e^{0.1671(\frac{N_{f}}{K_{f}})-0.5858}}\times \frac{e^{3.87637(\frac{N_{f}}{K_{f}})-5}}{1+e^{3.87637(\frac{N_{f}}{K_{f}})-5}}$	[68]
Offspring become feral from lack of exposure to humans	Free-roam to Feral	ff	0.05*	
Remain feral	Feral to Feral	1 –(fs)	0.9999	calculated
Surrender of feral	Feral to Shelter	fs	0.0001	

*only applies to offspring of free-roaming cats

^†^intact

^††^sterile

#### Owned cats’ transitions to other states

At each time step, owned animals have a certain probability of being relinquished (*r*), lost (*l*), abandoned (*a*), or of remaining in the owned state (Fig 1A). Cats are relinquished to shelters whereas cats that are lost or abandoned are functionally equivalent because they become unowned. We assumed a constant probability of 0.012 that cats will be relinquished (*r*) annually from data presented in New et al. [68].

Owned cats, especially indoor-outdoor cats, have a probability of being lost each year. However, it is important to consider the time scale of such events, consider lost pets in the demographic model as those that are lost over a one year time interval, and could only be returned via the shelter system. We use results from Weiss et al [69] who reported that 15% of cats were lost over a 5-year span. To estimate the annual loss of pets we used the equation

L=alog(t+1), (18)

set *t* = 5 and *L* = 0.15 and solved for *a*. Applying this equation, the annual probability of loss (where *t* = 1) is 0.058. While almost 6% of cats will get lost each year, the vast majority of these cats (75% in the case of [69]) will return to their owner or be returned by neighbors [69, 70]. To account for the vast majority of the cats returning home on their own we multiplied 0.058 by results from Lord et al. [70] who found 6 of 69 (0.086) lost cats were returned via the shelter system. The product of these two estimates was the annual probability of an owned cat being lost (*l* = 0.0049).

Abandonment is the intentional release of animals or the cessation of care. Estimated rates of abandonment are unavailable. Data from Finkler and Terkel [71] suggests abandonment is about five times more likely than relinquishment. Based on our estimate above with regard to relinquishment (*r* = 0.012), we conservatively assume a constant annual probability of abandonment of 0.05. Finally, the probability of an owned cat remaining as an owned cat is simply one minus the sum of the probabilities of being relinquished, lost, or abandoned.

#### Shelter cats’ transitions to other states

**Fig 7:**
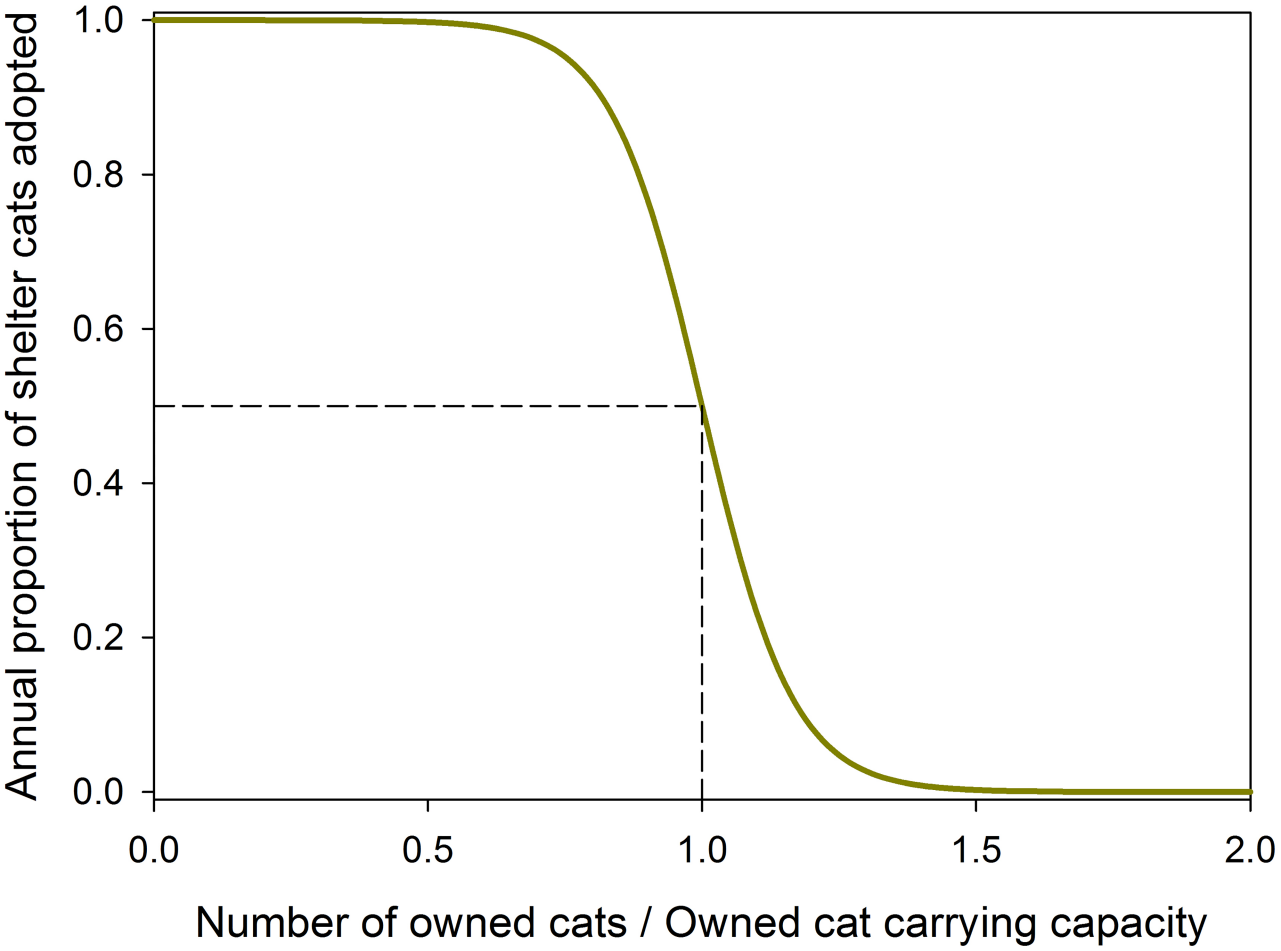
Proportion of shelter cats adopted. The annual proportion of cats being adopted from shelters was assumed dependent on owned cat carrying capacity following a 2-term logistic regression $\frac{e^{a\frac{N}{K}+b}}{1+e^{a\frac{N}{K}+b}}$ where a = -12, b = 12, N is the owned cat population size, and K is the owned cat carrying capacity. The reference line is the dashed line at the point where the probability of adoption is 0.5 and the population of owned cats is at carrying capacity.

At each time step, cats in shelters have a certain probability of transition via being adopted (*d*) or being reunited with an owner (*c*). The probability of remaining in a shelter was one minus the sum of the probabilities of being adopted or reunited with an owner (1-*d*+*c*). The probability of being adopted was assumed dependent on the carrying capacity of owned cats such that 50% of shelter cats are adopted when the owned population was at carrying capacity (Fig 7). This value corresponds with information from the Canadian Federation of Humane Societies [3] that reported that of the over 130,000 cats that entered shelters in Canada in 2012, 49.3% were adopted, 5.4% were reclaimed by owners, and 45.3% were euthanized. While there is little data on the probability of adoption relative to carrying capacity, we assumed that these statistics represent conditions at equilibrium, that is, when the number of owned cats was at carrying capacity. It should be noted that while this is a simplistic representation of the transition of animals out of the shelter, more complex models that include specific consideration of increasing capacity and decreasing length of stay [27] could be added for city-specific case studies.

The proportion of shelter cats being recovered by their owners was a function of sterilization status [70] and whether the cat has identifying information [72] as

$$recovery=(oy_{i}i)+\;(oy_{n}(1-i))$$ (19)

Here, *o* is the relative odds of recovery for intact compared to sterilized cats [70], *y* is the probability of return to owner for cats with (*y*_*i*_; 0.385) and without (*y*_*n*_; 0.018) identification [72], and *i* is the proportion of lost cats that carry identification (0.19; [70]: p. 217). The first part of the calculation accounts for cats with identification and the second half of the equation accounts for cats without identification. To calculate the relative odds of recovery for intact compared to sterilized cats (*o* = 0.25/0.57 = 0.4386), we used information from Lord et al. [70] who reported that sterilized cats (57%) were significantly more likely to be recovered by their owners than intact cats (25%).

#### Unowned cats’ transitions to other states

At each time step, unowned free-roaming cats have a certain probability of becoming owned by adoption off the street (*fa*) or under the stewardship of the shelter via surrender (*su*). The probability of remaining in the free-roaming cat category was one minus the sum of the probabilities of being adopted off the street, or surrendered to a shelter (1-*fa*-*su*).

**Fig 8:**
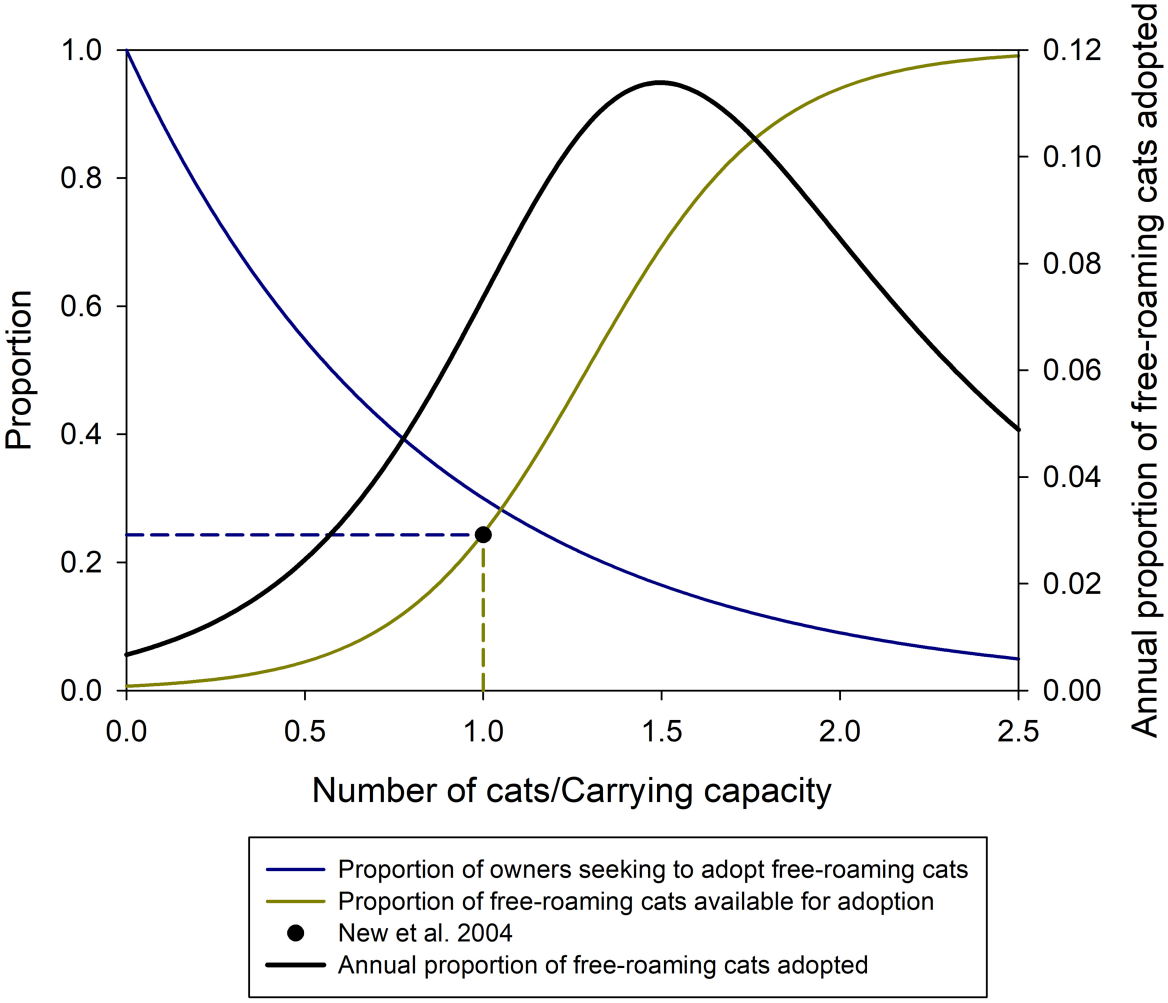
Proportion of free-roaming cats adopted off the street. The annual proportion of free-roaming cats adopted off the street (black line, right axis) was a product of two relationships: the proportion of cat owners that will seek to adopt a free-roaming cat and the proportion of free-roaming cats that are available to be adopted. The proportion of cat owners that will seek to adopt a free-roaming cat exponentially decreases as $e^{-a(\frac{N_{o}}{K_{o}})}$ where ***a*** = **1.20397**, ***N*_*o*_** is the number of owned cats and ***K*_*o*_** owned cat carrying capacity increases (blue line, left axis). The proportion of free-roaming cats that are available to be adopted (i.e. given temperament, personality and need for resources) is a logistic relationship of the form $\frac{e^{a(\frac{N_{f}}{K_{f}})+b}}{1+e^{a(\frac{N_{f}}{K_{f}})+b}}$ where ***a*** = **3.87637**, ***b*** = −**5**, ***N*_*f*_** is the number of free-roaming cats, and ***K*_*f*_** is free-roaming cats carrying capacity (yellow line, left axis). The dotted line is the value from New et al. [68] that found 0.243 of owned cats were acquired as strays and was used as a guide point to build the logistic function and assumes the system studied by New et al. [68] was at equilibrium (black dot).

We assume adoption of free-roaming cats is a density-dependent relationship that reflects the abundance of free-roaming cats in a community and the ratio of the number of owned cats to the carrying capacity of owned cats. The proportion of the free-roaming population that will be adopted off the street is the product of the proportion of owners that are looking to get a new cat and the availability of free-roaming cats that are suitable for adoption (i.e. are adoptable, given temperament, personality, and need for resources). We assumed that as the ratio of owned cats to carrying capacity increases, fewer cats will be adopted off the street as an exponential decay curve of the form *e*^−*a*(*N*/*K*)^ where the fraction is the ratio of owned cat population size (N) to its carrying capacity (K). We assumed at equilibrium that the proportion of owners that would adopt off the street would be 0.3 which resulted in *a* = 1.204. The availability of cats was defined using a two-parameter logistic function of the form

$$\frac{e^{ax+b}}{1+e^{ax+b}}$$ (20)

where we assumed a = -5 given a probability of very close to 0 when x = 0, and where x was the fraction of the ratio of the free-roaming cat population size to its carrying capacity. Using this equation, we applied findings from New et al. [68] who found the proportion of new cats acquired as strays was 0.243 which we assume occurred at equilibrium to solve for *b* = 3.876. The product of these two functions determines the proportion of free-roaming cats that are adopted off the street each year (Fig 8).

We expect the number of free-roaming cats to be surrendered to shelters to increase as free-roaming cat density increased but only within the confines of the carrying capacity of the shelter. In this way, our scenario reflects what many who work with cats are experiencing: increasing free-roaming cats and over-taxed animal shelters that are both at capacity for resources and spaces for cats [3, 73]. Over time, it also seems likely that kittens born to free-roaming cats could become unfit for living with humans due to lack of social contact with people so we assume a constant annual probability (*ff* = 0.05) of this occurring to kittens born to free-roaming females.

We assume feral cats remain feral except for a very small annual probability of being captured and surrendered to shelters (*fs* = 0.0001). In many cases these cats are eventually euthanized because they cannot be rehomed. The probability of remaining a feral cat was one minus the probability of being surrendered to a shelter.

### Model analysis

#### Population abundance

We started each simulation with an arbitrary starting cat population for intact juveniles, intact adults, sterile juveniles, and sterile adults for owned (100 intact juveniles, 100 intact adults, 100 sterile juveniles, 100 sterile adults), shelter (0, 0, 0, 0), free-roaming (300, 300, 300, 300), and feral (40, 40, 40, 40). We initiated the model to run for a maximum of 1200 iterations (years) until the cat population reached a steady state equilibrium. Once at equilibrium, we extracted the population abundance of all stages from each subpopulation, the vital rates, and carrying capacity. For our case study, we conducted a perturbation analysis to calculate relative sensitivity (elasticities) values for each subpopulation and life stage (see below).

#### Sensitivity analysis

We estimated the transient elasticities of population abundance to perturbation of the transition and demographic vital rates within the periodic matrix [28, 43]. The matrix calculus approach [43, 49] was used to allow transient elasticities of each variable to be summarized across life stages, subpopulations, or categories of reproductive status; in other words, we can understand how any perturbation, at any point in the system, influences population abundance across the network. This is critical to fully assess how human interventions aimed at addressing cat population dynamics have an influence on all sectors of the cat population. Following Caswell and Shyu [43], sensitivity of the asymptotic population vector **n** with respect to all lower-level parameters in the vector *θ* is:

$$\frac{dn}{{d\theta }^{T}}=\frac{dn}{{dvec}^{T}A}\frac{dvecA}{{d\theta }^{T}}$$ (21)

where *d*vec**A** is the vectorized derivative of the periodic product matrix **A** and ^T^ is the transpose. The elasticity of **n** with respect to *θ* is then:

$$\frac{\epsilon n}{{\epsilon \theta }^{T}}={D(n)}^{-1}\frac{dn}{{dvec}^{T}A}D(\theta )$$ (22)

where D(x) is a diagonal matrix with *x* on the diagonal and zeros elsewhere [43]. Summing the values of *ϵ****n***/*ϵθ*^*T*^. among subpopulations, vital rates or transition parameters allows the ability to assess the relative sensitivity of total population abundance to specific demographic rates that may be the focus of interventions by stakeholders. For example, the relative sensitivity of total cat abundance to the adoption rate in shelters. If the focal population is only a single cohort of the total population (e.g. only immature, unowned cats), then applying *D*(*x*) in (22) as a matrix of 1’s in those positions along the diagonal and all other entries being 0’s, allows us to derive the elasticity of immature, unowned cat population abundance to demographic vital rates among any subpopulation, vital rate or transition parameter across the entire network. For example, the relative sensitivity of total abundance of immature, unowned cats to sterilization of unowned cats (e.g. TNR) or, in contrast, the relative sensitivity of total abundance of immature, unowned cats to sterilization of owned cats (e.g. those performed by owners of their owned cats). In this manner, we can consider the sensitivity of each subpopulation, or portion thereof, individually or collectively depending upon the population of interest. For example, shelter workers are likely interested in the dynamics that influence shelter cat abundance while ecologists interested in the impacts of outdoor cats on wildlife populations are most likely interested in the dynamics that influence outdoor cat (outdoor owned, free-roaming, and feral) population abundance. Sensitivities can show contrasting effects on population abundances of different age/stages across the network and thus are of primary interest to managers to make effective management decisions [74].

#### Model validation

We used two different data sets from studies that provide population-level metrics that could be compared to those predicted by our population model to assess the model fit. First, we assessed the relationship between the unowned cat population abundance (sum of free-roaming and feral cat categories) from the model to city-specific estimates of “feral” cats provided by Blancher [5] for seven major cities in Canada. Blancher [5] provided mean feral cat population estimates, and for a subset of cities maximum and minimum estimates, from expert opinion. Second, we assessed the relationship between the model-predicted proportion of the feral cat population that was sterilized to the proportion of sterilized and intact cats admitted to intensive TNR programs for seven USA cities [42]. From the counts presented in Wallace and Levy [42], we calculated the binomial 95% CI of the proportion of the unowned cat population that was sterile in each city.

For each focal city examined, we extracted urban area, number of dwellings, and location (latitude) from Canada [55, 56] or USA [57, 58] census data to use as inputs for the population model and using the parameters as outlined above (Tables 1, 3 and 4). The model failed to converge for Honolulu, Hawaii likely owing to the nearly year-round breeding at southern latitudes in this population. Using the remaining six cities, we extracted from the model output the population abundance for each stage and state to derive the model-predicted outputs to compare to the field data provided in Blancher [5] and Wallace and Levy [42]. We assessed the fit of these two data sets by using linear regression models to test if the intercept was significantly different from 0 and the slope was significantly different from 1. In the plotted results, we also added a 1:1 line where points that fall on the line would suggest a perfect fit between model-predictions and estimates derived above.

**Table 4:** Carrying capacity of female cats in an urban area assuming 1:1 sex ratio in the population. In the table, the symbols represent: *H*—number of households in urban area, *Hc*—proportion of households with one or more cats, *Ch*—average number of cats per household with one or more cats. *C* is shelter capacity and *S* is average length of stay. *D*_*f*_ is log-transformed density of free-roaming cats (cats/ha), *D*_*feral*_ is the transformed density of feral cats (cats/ha), city area is the area in hectares of the urban area of interest.

State	Value	Source
Owned cats	$\left(\frac{H\times Hc\times Ch}{2}\right)$	[3]
Shelter cats	$\frac{C\times (365/S)}{2}$	[3]
Free-roaming cats	$\frac{e^{D_{f}}\times city\;area}{2}$	S1 Table
Feral cats	$\frac{e^{D_{feral}}\times city\;area}{2}$	S1 Table

#### Model application case study: Guelph, Ontario, Canada

In a final exercise, we present a case study to demonstrate the possible uses of this model for municipalities that are interested in understanding the population dynamics of cats. Guelph, Ontario, Canada (model inputs from 2011 census data: latitude = 43.55°N, number of dwellings = 52620, urban area = 7885 ha) is a city of approximately 120,000 people comprised of a small downtown and a university campus with an enrolment of approximately 25,000 students. We used Guelph as a case study because previous work had estimated the population size of different segments of the cat population: owned, indoor/outdoor [26], outdoor (termed ‘free-roaming’ by [23]), and shelter intake statistics [27]. A local coalition of stakeholders known as the Guelph Cat Population Taskforce (http://www.guelphcats.org/) has worked together over the past four years to identify the issues surrounding community cats, conduct research to estimate cat population size, and promoted different stakeholders to work towards a collective cause of improving animal welfare and reducing the environmental impacts of cats in Guelph. We ran the model and made slight adjustments to the carrying capacity for unowned cats (free-roaming and feral) iteratively until there was correspondence between estimated population sizes at equilibrium of all subpopulation and previously published estimates of these subpopulation.

We used the model predicted estimates to plot several comparisons to empirical estimates to demonstrate the status of the cat population. For example, the model predicted estimate of owned cats was subdivided by the mean estimated proportion of Guelph households that allow outdoor access for their cats (40%;[26]) to plot indoor-only the indoor-only component of the owned cat population while total outdoor cats was the sum of owned indoor-outdoor, free-roaming, and feral cats. Given the equilibrium cat population abundance in Guelph, we then used transient sensitivity analysis to identify the demographic vital rates and transition probabilities that contributed to owned cat population abundance, shelter cat population abundance, unowned cat population abundance, and total cat population abundance [43, 49, 74].

## Results

### Model fit

**Fig 9:**
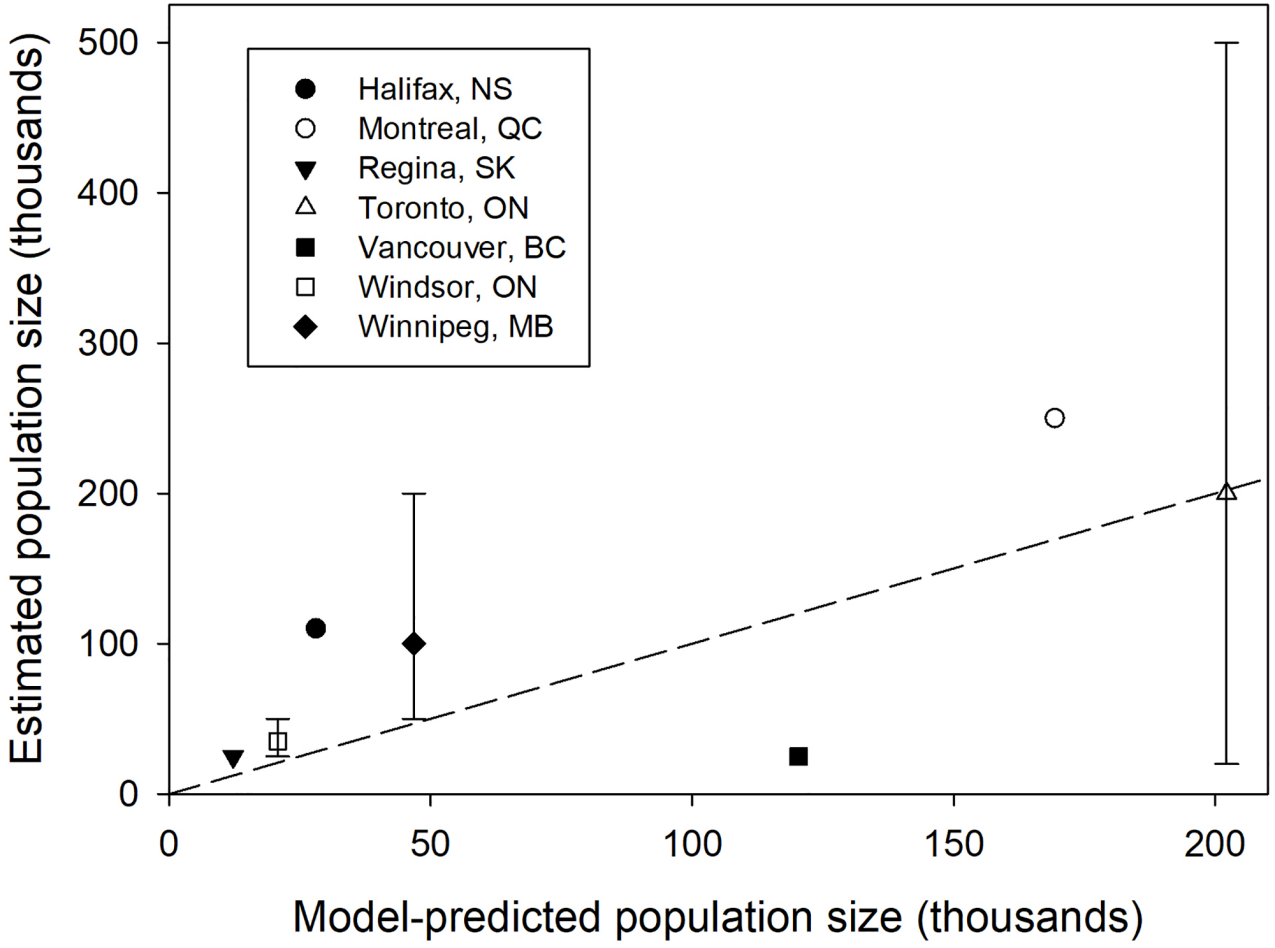
Relationship of cat abundance between model predictions and published estimates. Model-predicted population abundance of unowned cats (sum of free-roaming and feral cats) plotted against the expert-derived estimates of the abundance of unowned/feral/community cats in seven cities in Canada [5]. The error bars represent the minimum and maximum estimate in instances where more than one expert provided an estimate of the number of cats in a city. The female-only population estimate from the model was doubled to present as a total cat abundance which assumes a 1:1 sex ratio. The dashed line is the 1:1 relationship of agreement between the model and expert opinion.

In the model estimating the cat population size for seven Canadian cities, the intercept (t = 0.487, p = 0.65) and slope (t = -1.36, p = 0.23) were not significantly different from 0 and 1, respectively, indicating that the model-predicted unowned (free-roaming and feral) cat population size aligned with the feral cat population estimates in Blancher [5] (Fig 9). One of three population estimates that included error bars fell within the confidence intervals for feral cats with the other two being slightly below (Windsor = 4220 fewer cats estimated, Winnipeg = 3202 fewer cats estimated) the estimated population size based on expert opinion (Fig 9).

**Fig 10:**
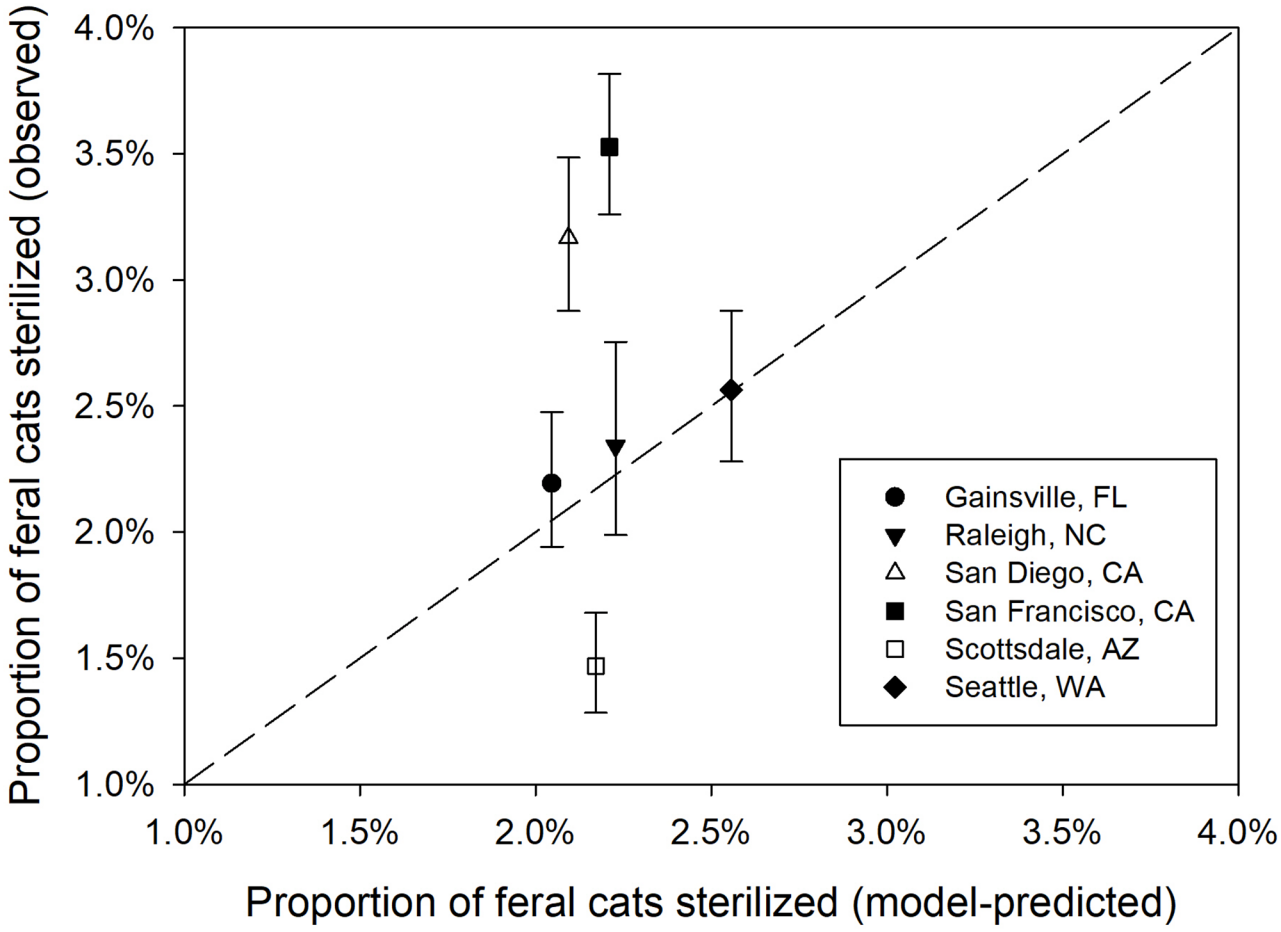
Relationship of cat sterilization rates between model predictions and published estimates. Model-predicted proportion of the population of feral cats plotted against the observed proportion of sterilized feral cats submitted to trap-neuter-return programs from for sterilization in six U.S. cities from [42]. The error bars are the binomial 95% CI of the sterilization proportion of the population in each city. Wallace and Levy [42] also provided data for Honolulu, HI but the model did not converge for that city. The dashed line is the 1:1 relationship of agreement between the observed and predicted values.

For the model predicting the proportion of sterile cats in six USA cities, there was little variation among the cities in the model-predicted proportion of sterile feral cats (Fig 10). The intercept (t = 6.77, p = 0.002) and slope (t = -8.04, p = 0.001) were significantly different from an intercept of 0 and a slope of 1, respectively, indicating that the model-predicted proportion of sterile feral cats did not match the observed proportion of sterile cats for the six USA cities. Although three of the estimates were well predicted, observed values and confidence intervals did not overlap with the expected values for the other three cities (Fig 10).

### Model application

**Fig 11:**
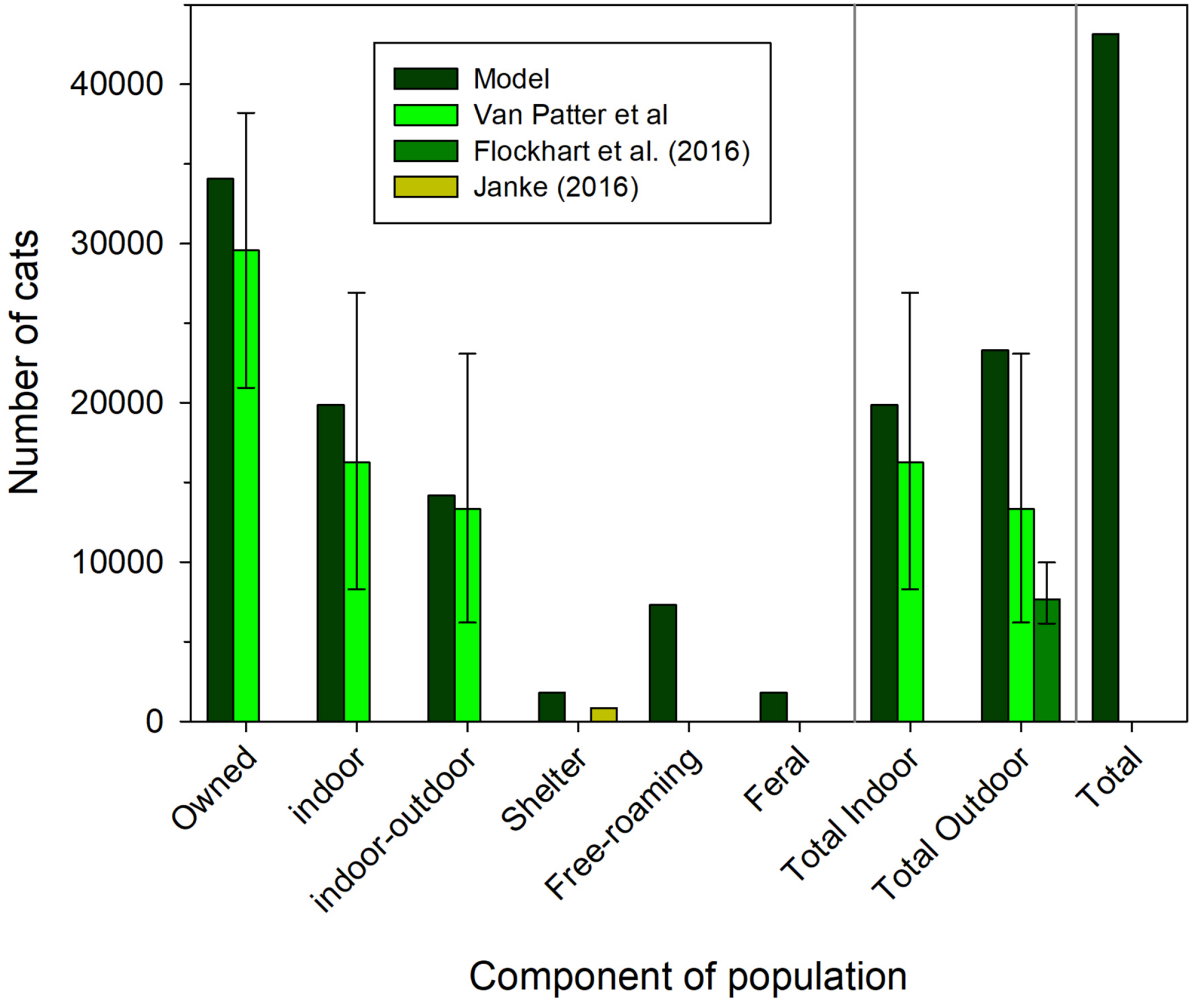
Population abundances of cats in Guelph, Ontario, Canada. The model-predicted numbers of cats (dark green bars) based on the four states of the model: Owned, Shelter, Free-roaming, and Feral. The owned cats are subdivided into indoor cats and indoor-outdoor cats based on the proportion of cats that have outdoor access (40%;[26]). The predicted total indoor cats is the number of indoor-outdoor cats and the total predicted outdoor cats is the sum of indoor-outdoor cats, free-roaming, and feral cats. The predicted total cat population is the sum of owned, shelter, free-roaming, and feral cats. The estimated mean and 95% confidence interval of the owned cat population abundance from Van Patter et al. [26] (light green bars) are based on 115 random surveys of Guelph residents. The estimated annual intake of cats into the Guelph Humane Society (yellow bars) is based on Janke [27] who reported 3295 cat intakes between 2011 and 2015. The estimated mean and 95% confidence interval of outdoor cat population abundance from Flockhart et al [23] (medium green bars) are based on a spatially explicit estimate based upon count data of free-roaming cats in Guelph in 2014. The model outputs are the number of female cats so we assume a 1:1 sex ratio in the population to show total population abundance which is what is reported in [23, 26, 27].

Using the starting parameter values of our model, free-roaming and feral cat subpopulations were smaller at equilibrium than those estimated using field data so we adjusted the carrying capacity of free-roaming (from 0.471 cats/ha to 0.787 cats/ha; or equivalently on the log-scale from *e*^-0.752664^ to *e*^-0.2400865^) and feral cat (from 0.079 cats/ha to 0.246 cats/ha; or equivalently on the log-scale from *e*^-1.400468^ to *e*^-2.536883^) subpopulations. Under these adjusted values, at equilibrium the cat population model predicted that 1,814 cats moved through the shelter system annually and there were 34,064 owned cats, 7,312 free-roaming cats and 1,782 feral cats in Guelph, Ontario, Canada (Fig 11). Overall, the predicted owned cat population abundance was within 95% confidence intervals of the estimated owned cat population abundance (Fig 11;[26]). The model predicted owned indoor-only cat and owned indoor-outdoor cat abundances aligned with empirical estimates [26]. The model predicted more cats moved through the shelter system annually than the approximately 824 individuals per year observed by Janke [27]. Total outdoor cat abundance included indoor-outdoor, free-roaming, and feral cats and the model predicted abundance was higher than estimates of Van Patter et al. [26] and Flockhart et al. [23]. The total population in Guelph was predicted to be 43,158 cats.

**Fig 12:**
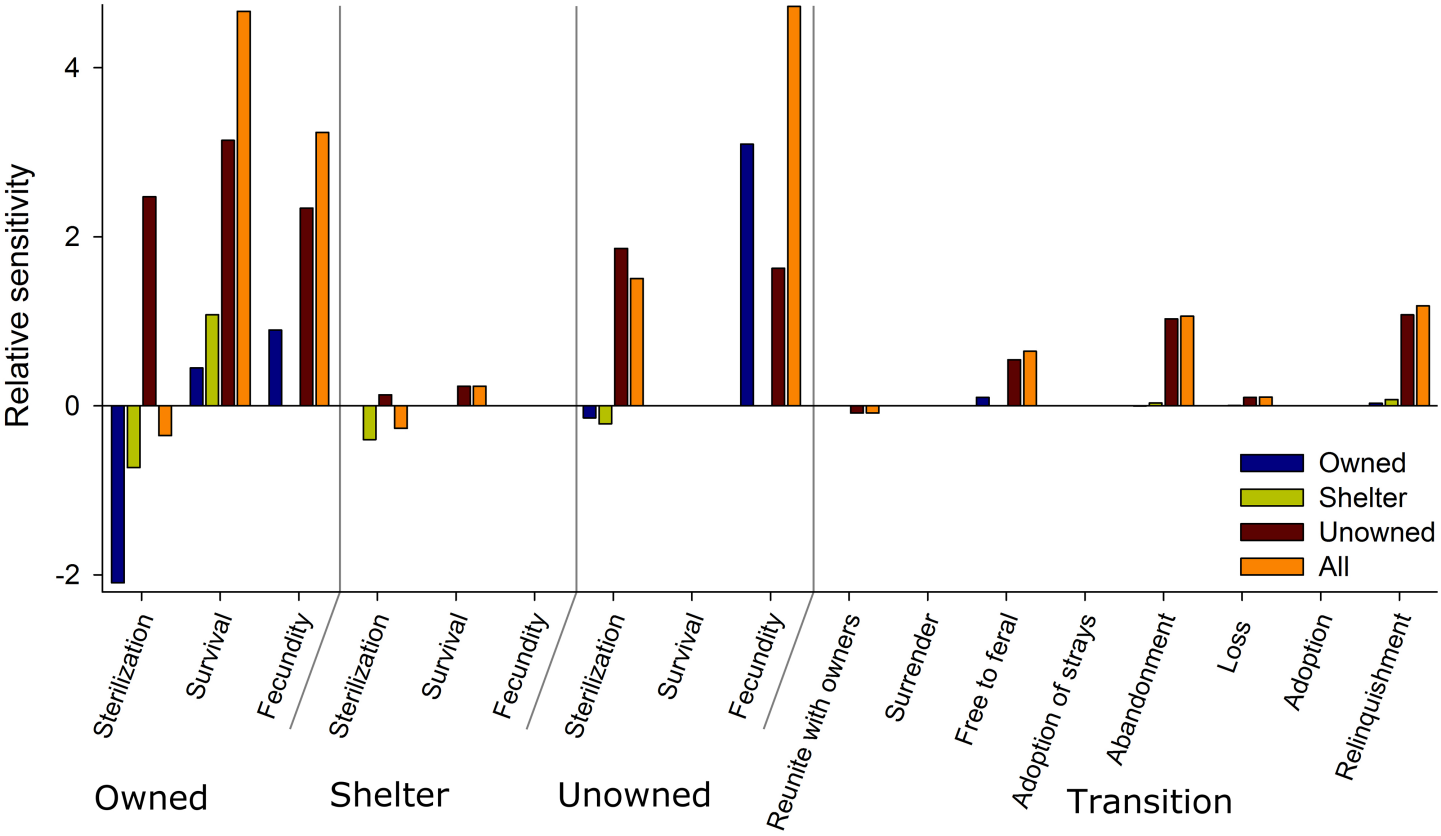
Relative sensitivities (elasticities) of cat population abundance in Guelph, Ontario to transitions and demographic vital rates. Sensitivities are depicted for owned (blue), shelter (green), unowned (red; sum of free-roaming and feral cats), and total cat abundance (orange) for each demographic rate and transition probability. Starting with the left most set of four bars, the values can be interpreted, as the sterilization rate of owned cats *increases*: the abundance of owned cats *decreases* (left-most blue bar), the abundance of shelter cats *decreases* (left-most yellow bar), the abundance of unowned cats *increases* (left-most red bar), and the abundance of total cats *decreases* (left-most orange bar).

The relative sensitivities of population abundance varied by demographic vital rate and transition probabilities among subpopulations across the network (Fig 12). The abundance of owned cats was most sensitive to changes in the fertility of unowned cats (increasing fecundity of unowned cats increased population abundance of owned cats) and owned cat sterilization rates (increasing sterilization rates of owned cats decreased population abundance of owned cats). The abundance of owned cats was not sensitive to changes in vital rates in shelters and was little affected by transition probabilities (Fig 12).

The abundance of cats in shelters was most sensitive to changes in demographic vital rates of owned cats and little affected by transition probabilities. The model predicts that the abundance of shelter cats should decrease with increasing sterilization rates across all subpopulations (Fig 12) suggesting a positive feedback between this intervention and reducing the probability of shelters being at or near capacity. The model predicted that increasing the sterilization rate of unowned cats should lead to a decrease in the abundance of cats in shelters which aligns with research conducted in Florida [75].

The abundance of unowned cats was sensitive to a large number of vital rates and transition probabilities (Fig 12). The unowned cat population was most sensitive to changes in the vital rates of owned cats and in all instances increasing these rates within the model demonstrated a predicted increase in unowned cat abundance. For example, increasing the sterilization rates of owned cats was predicted, by the model, to increase the abundance of unowned cats which represents a potential trade-off: sterilizing owned cats drives owned and shelter cat populations down but drives the unowned cat population up. The trade-off may arise from the network of the model structure because lost, sterilized cats have higher recovery rates, returned owned cats keep owned cat populations high near carrying capacity thereby reducing the number of unowned cats that are adopted off the street. Increasing the sterilization rate of unowned cats is predicted to increase the population abundance of this subpopulation (i.e., unowned cats). While this relationship may be the result of an assumed enhanced survival rate of sterile animals [34] in large unowned populations of cats such as those in Guelph (predicted 9,094 unowned individuals), it is unknown whether these population responses would be similar in a smaller population of cats. Increasing transition probabilities had consequences for the abundance of unowned cats. Specifically, unowned cats were predicted to increase with increasing probabilities of free-roaming kittens becoming feral, as well as both abandonment and relinquishment of owned cats (Fig 12).

Total cat abundance was most sensitive, based on the current model, to increasing fecundity of unowned cats and survival of owned cats and, to a lesser extent, the fecundity of owned cats (Fig 12). These results suggest that if our objectives are stated generally to manage for total cat abundance then interventions would best be focussed on reducing the fecundity of unowned cats but most of the potential numerical effects of this intervention would be seen in owned cat population abundance (Fig 12) given owned cats are the most abundant subpopulation (Fig 11).

## Discussion

Population estimates of cats in urban areas in North America from expert opinion [5] and empirical data [23, 26, 27] aligned with our model predicted population sizes of cats from different subpopulations. The model held less support for predicting population structure such as the proportion of cats within a subpopulation that are sterile as observed for a number of urban areas in North America [42]. Using perturbation analysis, we quantified the transient sensitivity of population abundance to all demographic vital rates and transition probabilities that arise due to human interventions for one city with reasonable estimates of the different subpopulations. While model predictions of population abundance highlight the magnitude of the challenge to humanely manage cats in urban areas, the sensitivity analysis helps to identify the scope of the resources and cooperation that will be necessary to find effective interventions to reach management objectives. Using three simple inputs available for any city in North America, our model provides a starting point to develop empirically-based, city-specific predictive population estimates. Future work to build off these ideas will require detailed data collection, parameter estimation, model structure refinement, and stochastic simulations to inform optimal management strategies. Collectively, quantitative models are one important tool that will complement other approaches, such as mental models and structured decision-making, to help inform management solutions across an entire urban network of cats.

It has long been recognized that cats may move between subpopulations during their life cycle [1], yet few population models exist for cats that account for these population transitions. Such models require a large number of parameters from all cat subpopulations including demographic vital rates and the transition of cats among the subpopulations [24]. Many of these vital rates (survival, reproduction, and sterilization) require intensive field data collection efforts, are difficult to measure (e.g. survival; [37, 38]), and may require stakeholder collaboration. Instead, most population models have considered only one portion of the population [13, 15, 16, 18, 19, 33] and most of these efforts have been focused on a limited number of specific interventions that influence population dynamics [15, 16, 18, 19] or developing specific management strategies [17]. Population models that ignore the transition of cats among subpopulations may provide less reliable information about the cat abundance and the impact on population dynamics of cats across a community.

We have presented a deterministic model that requires city-specific inputs (location, area, number of dwellings) and uses summary vital rates assumed or estimated from the literature. In that sense, the model presented here stands as a framework for municipal areas to integrate multiple data collection schemes into a single holistic population model. In its current form the model is not ready for immediate use. Rather, the model provides insight to stakeholders of the diversity of data necessary to build such a model and guidance to which data are the highest priority for developing a refined stochastic population model to inform management strategies of cats in a specific urban area. The Guelph Cat Population Taskforce provides an example of this type of effort in our community to gauge the human perceptions around cats and the outputs of our model predicts the number of cats, albeit with wide confidence intervals on empirically-based population estimates [23, 26]. Given the congruence between observed and expected population sizes, with ongoing refinement, a stochastic population model could be used to consider the optimal strategy to address cat overpopulation in that community. Developing these exercises in multiple urban areas should be a priority to facilitate communication and partnerships among those interested in cat populations, develop informed working models to predict cat population processes, and supply information to guide the development of humane and effective intervention programs to manage cats.

Models to date, formulated to achieve the specific objective of reducing unowned cat population growth, have concentrated on a small number of interventions focused on unowned cats—primarily euthanasia, trap-neuter-return and trap-vasectomy-hysterectomy-return [16, 18, 19]. Once refined for a specific geographic area, a stochastic version of the model that we propose could be used to consider multiple interventions concurrently to reach multiple objectives that may collectively have larger impacts on cat population dynamics across a community. For example, actions focused on owned cats might include subsidized spay-neuter programs [76, 77], campaigns to reduce the proportion of owned cats given outdoor access [78], and techniques that increase relinquishment over abandonment. Similarly, actions considered in population model simulations focused at cats in the shelter may include assurance that all cats moving through shelters are sterilized, that animal adopters are optimally matched with adoptable pets to reduce subsequent relinquishment rates [79], and techniques to increase reuniting lost animals with their owners [72]. For unowned cats, simulations may consider the relative impact of euthanasia of free-roaming cats compared to feral cats under the assumption that cats must first be captured to be euthanized and capture rates are density-dependent [80]. Ultimately, increasing the potential number of interventions to manage cats—and considering the subpopulation where those interventions are implemented—may provide an ability to identify less divisive management strategies that reach societal objectives by both considering humane interventions and minimizing the environmental impacts of outdoor cats.

Population models can be used to guide and develop management strategies for cats by capturing the natural variation and uncertainty around population dynamics. Natural variation in environmental conditions can lead to population fluctuations. For cats, these include typical environmental conditions that influence outdoor cat populations such as weather [31] as well as variable human-related conditions such the economy that influence factors such as disposable income that influence population dynamics of companion animals [32]. Given that we did not consider uncertainty in parameter estimates or stochastic variation inherent in population dynamics, future work aimed at developing robust strategies to manage cats would need to build off our model in two ways. First, stochastic population models should be developed to capture variance in population dynamics and to understand the probability of certain population outcomes. For example, given natural fluctuations in population abundance, what is the probability of a population decline due to natural factors over a given time frame and how do these baseline conditions serve to compare to the potential effects of actions aimed to deliberately decrease population size. These comparisons are necessary to better realize the potential outcomes of management strategies, avoid misleading expectations of different management actions, and provide decision-makers with the relative odds of a given strategy being successful of reaching a management objective. Second, stochastic population models should be paired with a decision model to determine optimal management sequences to reach cat population objectives for specific urban areas in the face of uncertainty [81]. Such modeling efforts provide decision-makers an expectation of the resources (e.g. money) necessary to reach an objective by a certain date given the dynamic responses of populations to natural conditions and specific management actions. Given the magnitude of the data needed to develop and refine these models, it will require stakeholders to work together and to focus data collection for specific urban areas.

Analysis of population models provide information about how population dynamics may influence management decisions. The results of perturbation analysis identify which vital rates and transitions have the largest potential to influence population abundance. Managers should consider sensitivity values in two ways. First, sensitivity values identify the vital rates that have the largest influence on uncertainty in the model predictions. These values may arise from poor data used to estimate parameters used in the model and highlight where research efforts should be focused to improve the robustness of model predictions [30]. In the case of Guelph, high quality data should be collected to estimate the demographic rates of owned and unowned cats. Once the data underlying a sensitive parameter is of high quality, a secondary use of sensitivity values allow managers to identify which vital rates and transitions should be targeted to manage a given population [49, 74] and which interventions are the most cost-effective [82].

For our case-study of Guelph, all subpopulations had elasticity values indicating that targeting interventions toward specific vital rates or transition probabilities could contribute to lowering the abundance of a specific subpopulation. These results provide insight to stakeholders about how they might invest their resources to reach their objectives and, importantly, that interventions applied by any stakeholder on cats in other subpopulations may enhance the likelihood of reaching their objectives. For example, organizations or stakeholders interested in reducing animal-shelter intake and promoting successful outcomes might consider advocating for increasing the sterilization rate of owned and unowned cats in addition to continuing (or embracing) mandatory sterilization techniques within shelters. Stakeholders interested in reducing the number of unowned cats might consider ways to enhance the recovery of lost cats with their owners, decrease abandonment, and decrease relinquishment which was predicted to directly reduce the unowned cat population abundance. However, we note specifically that these findings are based on the outcome of a deterministic model that is at equilibrium and did not consider how any specific intervention would influence the dynamics of the cat population. Despite this, the complex relationships in such a connected network model suggest that interventions focused on any specific vital rate or transition probability will have complex repercussions across the entire cat population network. As a result, uncoordinated action plans among different stakeholder groups could be counter-productive to reaching multiple societal objectives, could collectively take more time, and ultimately cost more money.

### Limitations

Our model showed promise for better understanding cat population dynamics; however, there are several caveats that currently limit its widespread application. Few studies were available to estimate some of the demographic and transition rates that were applied in the model and, in many cases, vital rate estimates were provided in the literature without estimates of variance that reduce our confidence of their application in the model. On one hand, estimating vital rates such as survival and reproduction in the field are labor intensive, require long study periods, and only focus on one small section of the population [35, 38]. On the other hand, estimation of per capita transition rates was also limited, despite many studies presenting raw counts of cat intakes and outcomes [3, 59, 60], because we do not know the size of the focal cat population. While data limitations and parameter uncertainty is inherent to any population model, there clearly is an opportunity for ecologists and demographers to collaborate with stakeholders working directly with cats—shelters, veterinarians, municipal animal services, feral cat welfare advocates—to develop data collection approaches and statistical analyses to refine parameter estimates and functional relationships for a structured cat population model.

As evidenced by the model failing to converge using data from an urban area at a southern breeding latitude in Hawaii, our model is unlikely to be applicable to all urban areas and caution must be used when trying to extrapolate the predictions from the current model to other urban areas. Instead, stakeholders should work together to develop and refine a specific model that is structured in a way that is most useful for their community and for its intended purpose. For cats, this requires focused studies to estimate demographic vital rates, transition rates among subpopulations, and especially evidence for density dependence. Despite the challenges of data availability (e.g. carrying capacity) and uncertainty in the strength and shape of mechanistic feedbacks (e.g. density dependent survival of unowned cats and adoption rates of unowned cats), our model serves as a framework of how these research elements might fit together and provide initial hypotheses that should be tested with field data. Ideally, a number of cities would self-identify as case studies and the cities would best range across a gradient of urban area sizes and be chosen to fully capture spatial variation in demographic vital rates (e.g. survival, breeding season length), transition, carrying capacity estimates, and population size. In other words, the assumed functional relationships that we used should not be applied without first comparing to empirical data and, for model validation, robust estimates of cat abundance for each subpopulation. Despite the fundamental need for these metrics, few robust estimates of cat populations exist owing to the challenges associated with counting cats [23] which highlights a key research priority.

Unlike previous models [13–16, 18, 19], our model did not compare different management strategies for cats. While our model did calculate sensitivities of population size at equilibrium to demographic and transition rates, these represent the effects under small changes to the vital rates and therefore do not represent how the dynamics in a population would vary under different management strategies over time. Therefore, we cannot, without further refinement of a model for a specific location, define what inventions might be the most appropriate for a given urban area to reach specific population size objectives. Furthermore, we did not consider stochasticity in our model despite cat populations being known to vary across space and time [23, 31]. Accounting for uncertainty in demographic and transition rates is fundamental to derive robust model predictions on cat population size and develop management strategies for a given urban area.

## Conclusions

Cats are among the most popular pets in our homes and they provide significant benefits for owner welfare and companionship [83, 84]. Issues surrounding cat population density within a community are complex and are largely due to the actions (or inactions) of people. Cat population dynamics result from the demographic vital rates within and transition between three segments of the population: owned cats (indoor, indoor/outdoor), unowned cats (free-roaming, true feral), and cats in the shelter system. Management of cat population density must consider all portions of the population because human intervention greatly facilitates movement between the groups and each group contributes differently to total cat population growth. Population models that simultaneously predict population abundance across all subpopulations of cats within an urban area provide a tool to recognize the challenges and to engage stakeholders to work together to reach common or different objectives concurrently. [26]

## Supporting information

S1 Table Free-roaming and feral cat population density records from published literature. (CSV)
