# Correction: Multistate matrix population model to assess the contributions and impacts on population abundance of domestic cats in urban areas including owned cats, unowned cats, and cats in shelters

**DOI:** 10.1371/journal.pone.0245633

**Published:** 2021-01-13

**Authors:** D. T. Tyler Flockhart, Jason B. Coe

In the Methods, there is an error in the seventh equation of the demographic matrix ***B***_*i*_ of state *i*. The transitions from intact adult to altered juvenile and intact adult to intact juvenile use the term *n*_*1*_, when it should be *n*_*0*_. Please view the complete, correct equation here:

$$B_{i}=[\begin{array}{cccc} \sqrt{s_{0}}f_{0}(1-n_{0}) & \sqrt{s_{1}}f_{1}(1-n_{0}) & 0 & 0\\ s_{0}(1-n_{0}) & s_{1}(1-n_{1}) & 0 & 0\\ \sqrt{s_{0}}f_{0}n_{0} & \sqrt{s_{1}}f_{1}n_{0} & 0 & 0\\ s_{0}n_{0} & s_{1}n_{1} & s_{0} & s_{1} \end{array}]$$ (7)
